# Green Solvents for Eco-Friendly Synthesis of Dimethindene: A Forward-Looking Approach

**DOI:** 10.3390/molecules27217594

**Published:** 2022-11-05

**Authors:** Andrea Francesca Quivelli, Federico Vittorio Rossi, Chiara Alario, Francesco Sannicolò, Paola Vitale, Joaquín García-Álvarez, Filippo Maria Perna, Vito Capriati

**Affiliations:** 1 Dipartimento di Farmacia—Scienze del Farmaco, Università degli Studi di Bari “Aldo Moro”, Consorzio C.I.N.M.P.I.S., Via E. Orabona 4, I-70125 Bari, Italy; 2 Laboratori Alchemia S.r.l., Via San Faustino 68, I-20134 Milan, Italy; 3 Laboratorio de Química Sintética Sostenible (QuimSisSos), Departamento de Química Orgánica e Inorgánica, (IUQOEM), Centro de Innovación en Química Avanzada (ORFEO-CINQA), Facultad de Química, Julián Clavería, 8, 33006 Oviedo, Spain

**Keywords:** dimethindene, deep eutectic solvents, API synthesis, synthetic methodologies, green chemistry, cyclopentyl methyl ether, 2-Methyltetrahydrofuran

## Abstract

Dimethindene is a selective histamine H_1_ antagonist and is commercially available as a racemate. Upon analyzing the synthetic pathways currently available for the industrial preparation of dimethindene, we set up a sustainable approach for the synthesis of this drug, switching from petroleum-based volatile organic compounds (VOCs) to eco-friendly solvents, such as 2-methyltetrahydrofuran (2-MeTHF) and cyclopentyl methyl ether (CPME) belonging to classes 3 and 2, respectively. Beyond decreasing the environmental impact of the synthesis (E-factor: 24.1–54.9 with VOCs; 12.2–22.1 with 2-MeTHF or CPME), this switch also improved the overall yield of the process (from 10% with VOCs to 21–22% with 2-MeTHF or CPME) and remarkably simplified the manual operations, working under milder conditions. Typical metrics applied at the first and second pass, according to the CHEM21 metrics toolkit, were also calculated for the whole synthetic procedure of dimethindene, and the results were compared with those of the classical procedure.

## 1. Introduction

In recent years, growing environmental awareness has led chemists to consider the sustainability of a chemical process as a “key driver for innovation” in the pharmaceutical industry [1]. In order to develop greener and more effective methods to produce active pharmaceutical ingredients (APIs), Anastas and Warner’s 12 Principles of Green Chemistry became the guidelines for building an environmentally responsible manufacturing process [2,3]. Recently, the direct or accidental release of treated solvent waste and toxic chemicals into the environment, as well as hazardous work conditions, have led to the implementation of environmental directives and legislation (Clean Air Act of 1990 and the European Union Solvents Emission Directive 1999/13/EC) and the regulation of the usage of potentially harmful or environmentally damaging chemical substances (Registration, Evaluation, Authorisation, and Restriction of Chemicals (REACH)) [4]. These governmental regulations, in addition to many others, have created widespread interest in green chemistry and sustainable technology [5].

During pharmaceutical process development, a solvent selection analysis is pivotal for determining the sustainability of future production lines. Toxic and harmful volatile organic compounds (VOCs) (50–100 °C) (e.g., dichloromethane (DCM) and toluene), traditionally employed as solvents by organic and industrial chemists, have been severely curtailed and blacklisted. In this vein, the whole question of solvents requires rethinking and has become a primary focus, especially in the manufacture of pharmaceuticals because solvents contribute over 80% to the organic waste produced [6,7]. Not only is this “solvent problem” related to their use, but it also has implications with regard to their containment, recovery, and reuse. Thus, increasing attention has been paid to the efficient removal of solvents from manufactured drugs, with the solvents themselves also classified as impurities.

**Scheme 1:**
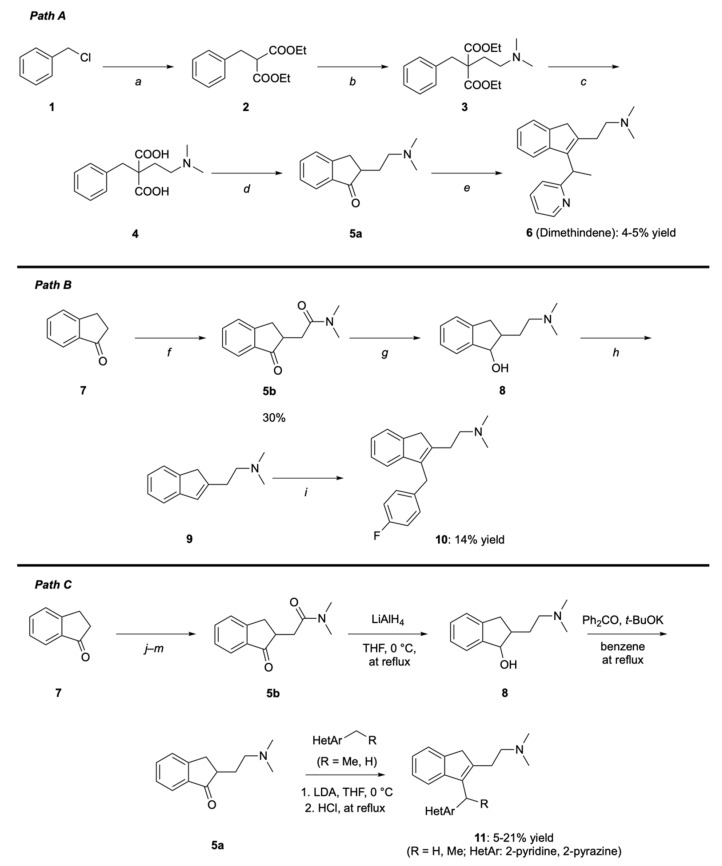
Current industrial synthesis of dimethindene (**6**) using VOCs: **Path** (**A**) [11,12]; synthesis of dimethindene analogs **10**,**11** using VOCs: **Paths** (**B**,**C**) [13,14]. (**Path A)** Reagents and conditions: (*a*) diethyl malonate, Na, and EtOH, at reflux. (*b*) NaH, 2-chloro-*N*,*N*-dimethylethan-1-amine, and toluene, at reflux. (*c*) NaOH and EtOH/water at reflux. (*d*) PPA, heating. (*e*) 2-Ethylpyridine, *n*-BuLi, and Et_2_O, at −78 °C; HCl (20%), at reflux. **Path** (**B**) Reagents and conditions: (*f*) LDA and THF, at −78 °C; 2-chloro-*N,N*-dimethylacetamide. (*g*) LiAlH_4_ and THF, at reflux. (*h*) AcOH and HCl, at reflux. (*i*) 4-Fluorobenzyl bromide, *n*-BuLi, and THF, at −78 °C. **Path** (**C**) Reagents and conditions: (*j*) glycolic acid (40% in H_2_O), conc. H_2_SO_4_, and dioxane, at reflux; (*k*) Zn, HOAc, and H_2_O, at 100 °C; (*l*) (COCl)_2_, cat. DMF, and DCM; (*m*) HNMe_2_, Et_3_N, and DCM.

Building on our interest in the use of environmentally responsible solvents in the synthesis of APIs [8,9], we became eager to develop, in cooperation with our industrial partner (Laboratori Alchemia s.r.l.) [10], a sustainable synthesis method for dimethindene (**6**) (Scheme 1), which is a first-generation selective H_1_-antihistamine drug and is commercially available as a racemate. It was patented in 1958 and was first introduced for medical use in the 1960s. Because of pressing requests from the Italian and European markets, the synthesis of this drug has attracted a lot of API manufacturers. Currently, the industrial synthesis of dimethindene involves the massive use of genotoxic benzyl chloride (**1**) and a large amount of polyphosphoric acid (PPA), which are both hazardous to the environment and human health (Scheme 1A) [11,12]. The process involves five steps. In the first one, diethyl malonate reacts with **1**, affording the benzyl malonic diethyl ester **2** (*a*), which is then alkylated with commercially available 2-chloro-*N*,*N*-dimethylethan-1-amine in toluene to yield the tertiary amine **3** (*b*). The subsequent saponification of **3** leads to the amino diacid **4** (*c*). This is followed by intramolecular Friedel–Craft acylation carried out in PPA to yield the indanone derivative **5a** (*d*). The reaction is finally quenched by cautious addition of ice chips and K_2_CO_3_, which causes a vigorous release of CO_2_. The treatment of **5a** with the lithium salt of the commercially available 2-ethylpyridine forms a tertiary alcohol in situ, whose dehydration in refluxing HCl (20%) produces dimethindene (**6**) (*e*). The overall yield of the process is around 4–5%.

Beaton and co-workers [13] synthesized the intermediate **5b** (30% yield) by preliminary treating a THF solution of 1-indanone (**7**) with LDA at –78 °C and then quenching the resulting enolate with 2-chloro-*N*,*N*-dimethylacetamide (*f*) (Scheme 1B). After the reduction of carbonyl groups of **5b** followed by acid-mediated dehydration of secondary alcohol **8** (*g*,*h*), *n*-BuLi and 4-fluorobenzyl bromide were sequentially added to a solution of **9** (*i*). The desired product **10** (a dimethindene analog) was isolated with an overall yield of 14%. In 2010, the same authors developed another synthetic approach to dimethindene analogs (Scheme 1C) [14]. In this case, **7** was first converted into **5b** by a four-step process involving condensation with glycolic acid (*j*) followed by reduction with Zn in acetic acid (*k*), acyl chloride formation catalyzed by dimethylformamide (DMF) in DCM (*l*), and reaction with dimethylamine in DCM (*m*). Simultaneous reduction of the amide and ketone moieties of **5b** with LiAlH_4_ led to alcohol **8**, which was oxidized to produce **5a**. Lithium salts from various alkyl heteroaryls, generated by the treatment of alkyl heteroarenes with LDA, were finally added to **5a**, followed by acid-mediated dehydration to afford dimethindene analogs **11**. Of note, among the various VOCs, experts particularly recommend avoiding dimethylacetamide and DMF, as they bear a risk of nitrosamine formation [15].

With the aim of minimizing the environmental impact of pharmaceutical processes, herein, we report a forward-looking approach for sustainable and more effective synthesis of dimethindene (**6**). Upon a careful solvent selection based on the replacement of VOCs with more environmentally friendly solvents, such as 2-MeTHF [16] and cyclopentyl methyl ether (CPME) [17], we were able to successfully modify the current industrial process (*vide infra*), not only decreasing the waste of solvents/materials but also improving the overall yield of the process, which increased from 10% in VOCs to 21–22% in 2-MeTHF/CPME.

## 2. Results and Discussion

### 2.1. Alternative Retrosynthetic Approaches to Dimethindene (***6***)

**Scheme 2:**
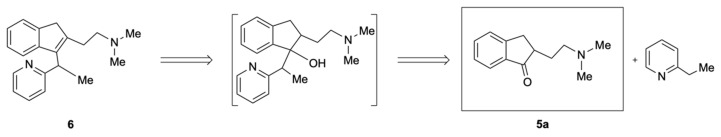
An alternative retrosynthetic approach to dimethindene (**6**).

Inspired by these results, the synthesis of dimethindene was then planned according to the retrosynthetic approach depicted in Scheme 2. It involved two different steps: (i) the addition of 2-ethylpyridine to the carbonyl moiety of **5a**, followed by (ii) the dehydration of the corresponding intermediate tertiary alcohol.

**Scheme 3:**
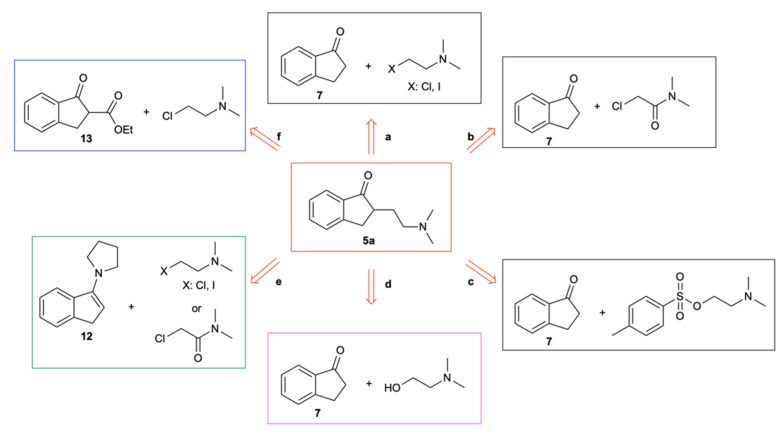
Possible retrosynthetic approaches to **5a**. (a) α-Functionalization of 1-indanone (**7**) with 2-chloro- or 2-iodo-*N*,*N*-dimethylethan-1-amine; base: LDA, KHMDS, and NaHMDS; solvent: THF and toluene; temperature: −78 °C or −20 °C; (b) α-functionalization of 1-indanone (**7**) with 2-chloro-*N*,*N*-dimethylacetamide; base: LDA and NaH; solvent: THF and CPME; temperature: −78 °C or 0 °C or 25 °C; (c) α-functionalization of 1-indanone (**7**) with 2-(dimethylamino)ethyl-4-methylbenzenesulfonate; base: LDA and NaH; solvent: THF, toluene, and CPME; temperature: −78 °C or −20 °C or 0 °C; (d) α-functionalization of 1-indanone (**7**) with 2-(dimethylamino)ethan-1-ol; Ru cat.: [Ru(*p*-cymene)Cl_2_]_2_; ligand: (oxydi-2,1-phenylene)bis(diphenylphosphine); solvent: toluene; base: CsCO_3_ and NaOH; temperature: 105 °C; 8 h; (e) reaction between enamine **12** and 2-chloro- or 2-iodo-*N*,*N*-dimethylethan-1-amine or 2-chloro-*N*,*N*-dimethylacetamide; solvent: ChCl/urea; temperature: 25 °C for up to 24 h; (f) reaction between β-ketoester **13** and 2-chloro-*N*,*N*-dimethylethan-1-amine; base: LDA, KHMDS, NaMDS, and NaH; solvent: THF, toluene, CPME, and DES; temperature: −78 °C, −20 °C, or 0 °C.

Several approaches were examined for the synthesis of **5a** (Scheme 3). The α-functionalization of 1-indanone (**7**) with different electrophiles (2-chloro- or 2-iodo-*N*,*N*-dimethylethan-1-amine, 2-chloro-*N*,*N*-dimethylacetamide, or 2-(dimethylamino)ethyl-4-methylbenzenesulfonate) in various conventional (e.g., THF, toluene, and CPME) and unconventional (e.g., the deep eutectic solvent (DES) choline chloride (ChCl)/urea (1:2 mol mol^−1^)) solvents [18,19,20,21] using different bases (LDA, KHMDS, NaHMDS, and NaH) proved to be ineffective, leading either to the recovery of the starting material (Scheme 3a,c) or to the isolation of **5a** at up to a 30% yield (Scheme 3b; base: LDA; solvent: THF or CPME) (see Supplementary Materials). Ru-catalyzed hydrogen-borrowing C–C bond formation [22,23] between **7** and 2-(dimethylamino)ethan-1-ol (Scheme 3d), the reaction between 2-chloro- or 2-iodo-*N*,*N*-dimethylethan-1-amine or 2-chloro-*N*,*N*-dimethylacetamide and enamine **12** (prepared in 93% yield from **7** and pyrrolidine in ChCl/urea; see Supplementary Materials) (Scheme 3e), and the reaction between 2-chloro-*N*,*N*-dimethylethan-1-amine and β-ketoester **13** (synthesized from **7** and diethyl carbonate in CPME; see Supplementary Materials) using different solvents (THF, toluene, CPME, and DES) and different bases (NaOH, CsCO_3_, LDA, KHMDS, NaHMDS, and NaH) (Scheme 3f) all led to a null yield of **5a**. These unsatisfactory results prompted us to pursue a different approach to dimethindene synthesis.

### 2.2. An Environmentally Sustainable Process for the Industrial Synthesis of Dimethindene (***6***)

A large amount of waste produced (80%) from the API manufacturing process is estimated to be solvent-related [24,25]. Thus, the selection, use, and recovery of solvents would contribute significantly to alleviating this problem. The shift toward greener solvents in the industrial synthesis of APIs is one of the main goals of pharmaceutical companies [26]. According to a recent ICH guidance Q3C (R8) [27], 2-MeTHF and CPME belong to solvent classes 3 and 2, respectively. Thus, they are recognized as “greener alternatives” to VOCs [28,29], and their use has been strongly advocated in the synthesis of drugs and drug intermediates by the ACS Green Chemistry Pharmaceutical Roundtable [30,31,32]. On this basis, we focused on the use of these solvents for the total synthesis of dimethindene (**6**). Moreover, to quantify the eco-sustainability of the synthesis of **6**, when using 2-MeTHF or CPME as the solvent, we made use of the First Pass CHEM21 Metrics Toolkit developed by Clark et al. [33,34,35], calculating atom economy (AE), reaction mass efficiency (RME), optimum efficiency (OE), effective mass yield (EM), and mass intensity (MI) of each step, along with process mass intensity (PMI) metrics, the latter taking into account the reactants, reagents, and solvents of the whole process (PMI_RXN_) or the solvents and reagents used in the work-up procedure (PMI_WU_), and we compared these values with the corresponding ones related to the classical synthetic procedure for **6** (see Supplementary Materials for details). In addition, some metrics typical of the Second Pass CHEM21 Metrics Toolkit, which quantify the use of material from renewable sources, such as renewables intensity (RI) and renewables percentage (RP), were also calculated.

The new approach followed for the synthesis of **6** comprised 4 steps and started from commercially available benzyl malonic diethyl ester **2** (Table 1). The latter (1.0 g, 4 mmol) was added to a refluxing suspension of NaH (1 equiv) in CPME or 2-MeTHF (10 mL each). After 1 h, 2-chloro-*N,N*-dimethylethan-1-amine (1 equiv) was added, and the reaction was refluxed for an additional 6 h. Compound **3** could finally be isolated at an 80% yield from 2-MeTHF and a 90% yield from CPME. Conversely, the yield of **3** was only 63% when using toluene as the solvent and 2 equiv of amine (Table 1, step 1). This allowed a significant improvement in reaction efficiency indicators, such as RME, OE, and E factor (Table 1, step 1). Furthermore, the use of CPME or 2-MeTHF as the reaction solvent and for the work-up step decreased the mass of non-benign reagents (and thus the indicator EM) while improving the recyclability of the process (RI and RP indicators).

The saponification of malonic ester **3** with an aqueous NaOH solution furnished the amino diacid **4** at a 75% yield, and the E factor for this step was very low (9.8). This reaction step was also characterized by a good RP value (90.1%) (Table 1, step 2). The ring closure reaction (Friedel–Craft acylation) to produce the indanone skeleton is generally carried out using an excess of PPA (which also acts as a reaction medium), affording **5a** at only a 20% yield. Conversely, a stoichiometric amount of PPA (1 equiv) in CPME or 2-MeTHF was enough to isolate **5a** at a 50% yield in CPME and a 55% yield in 2-MeTHF, with a safer and operationally easier work-up procedure, thereby decreasing the amount of mass of non-benign reagents (higher EM value), improving the efficiency of the reaction (higher RME and OE values), and reducing the MI_WU_ to a third compared with that of the classic procedure. The RP indicator for both reactions run in CPME and 2-MeTHF was very high (87–91.8%) (Table 1, step 3).

Finally, by carrying out the nucleophilic addition of the benzylic-type anion of 2-ethylpyridine to the carbonyl moiety of **5a** in CPME or 2-MeTHF at 0 °C, followed by dehydration of the corresponding tertiary alcohol in refluxing HCl, dimethindene (**6**) was isolated at a 65% yield, regardless of whether CPME or 2-MeTHF was used as the solvent. On the other hand, the employment of a VOC, such as Et_2_O, as an alternative solvent, required the cooling of the system to −78 °C and led to the isolation of **6** at a 60% yield (Table 1, step 4). Again, the use of green solvents improved the efficiency and the sustainability of the reaction as results of the quantitative metrics calculated, particularly for the EM indicator (Table 1, step 4).

In summary, the use of eco-sustainable solvents, such as CPME and 2-MeTHF, made it possible to improve the efficiency and sustainability of the entire synthetic process of dimethindene (**6**). The overall yield of the isolated product was twice that of the yield obtained through the current synthetic procedure using VOCs, with improvements in all quantitative metrics, especially the RP indicator (Table 2). Furthermore, the use of CPME or 2-MeTHF as the solvent allowed the reaction to be carried out under milder conditions (step 4) and avoided the use of toxic reagents in excess (Table 1, steps 1 and 3).

## 3. Materials and Methods

### 3.1. General Methods

The deep eutectic solvent ChCl/urea (1:2 mol/mol) was prepared by heating under stirring at 60–80 °C for 10–30 min the corresponding individual components until a clear solution was obtained. For ^1^H NMR (600 MHz) and ^13^C NMR (150 MHz), CDCl_3_ or D_2_O was used as the solvent; chemical shifts are reported in parts per million (δ). FT-IR spectra were recorded on a Perkin–Elmer 681 spectrometer. GC analyses were performed on an HP 6890 model Series II using an HP1 column (methyl siloxane; 30 m × 0.32 mm × 0.25 μm film thickness). Analytical thin-layer chromatography (TLC) was carried out on precoated 0.25 mm thick plates of a Kieselgel 60 F; visualization was accomplished by UV light (254 nm) or by spraying a solution of 5% (*w*/*v*) ammonium molybdate and 0.2% (*w*/*v*) cerium(III) sulfate in 100 mL 17.6% (*w*/*v*) aq. sulfuric acid and heating to 473 K until blue spots appeared. Chromatography was run using silica gel 60 with a particle size distribution of 40–63 μm and 230–400 ASTM. GC–MS analyses were performed on an HP 5995C model. Cyclopentyl methyl ether (CPME) was provided by Zeon Europe GmbH. High-resolution mass spectrometry (HRMS) analyses were performed using a Bruker microTOF QII mass spectrometer equipped with an electrospray ion source (ESI). Other reagents and solvents, unless otherwise specified, were purchased from Sigma-Aldrich (Sigma-Aldrich, St. Louis, MO, USA) and were used without further purification. The following abbreviations are used to explain the multiplicities: s = singlet, d = doublet, t = triplet, m = multiplet, br = broad, dd = double doublet. RT = room temperature.

### 3.2. Synthesis of Dimethindene ***6*** in Green Solvents

#### 3.2.1. Step 1: Synthesis and Characterization Data of 2-Benzyl-2-[2-(Dimethylamino)ethyl]malonic Acid Diethyl Ester (**3**)

To a refluxing suspension of NaH (0.096 g, 4 mmol, 1 equiv) in CPME or 2-MeTHF (10 mL), 2-benzylmalonic acid diethyl ester **2** (1.0 g, 4 mmol, 1 equiv) was added dropwise. The reaction mixture was refluxed for 1 h to yield a clear yellow solution. After this time, 2-chloro-*N,N*-dimethylethan-1-amine (0.43 g, 4 mmol, 1 equiv) was added dropwise, and the reaction mixture was refluxed for 6 h. The resulting suspension was extracted with HCl (5%) (3 × 1 mL). The aqueous layers were combined, basified with NH_4_OH (pH = 11–12), and extracted with CPME or 2-MeTHF (3 × 2 mL). The combined organic layers were dried (Na_2_SO_4_) and concentrated to produce compound **3** as a yellow oil at a 90% yield (1.157 g) in CPME and an 80% yield (1.028 g) in 2-MeTHF.

*2-Benzyl-2-[2-(dimethylamino)ethyl]malonic acid diethyl ester* (**3**): ^1^H NMR (600 MHz, CDCl_3_) δ 1.24 (t, *J* = 7.3 Hz, 6 H), 1.95–1.99 (m, 2 H), 2.21 (s, 6 H), 2.31–2.35 (m, 2 H), 3.24 (s, 2 H), 4.17 (q, *J* = 7.3 Hz, 4 H), 7.09–7.11 (m, 2 H), 7.20–7.26 (m, 3 H); ^13^C NMR (150 MHz, CDCl_3_) δ 13.9, 29.9, 38.6, 45.4, 54.8, 57.7, 61.1, 126.9, 128.2, 129.9, 136.2, 171.1; \FT-IR (film, cm^−1^): 2835, 1730, 1581, 1393, 1355, 1285, 1298, 1048, 900, 854, 762, 686; GC/MS (70 eV) *m*/*z* (%): 321 (M^+^, 100), 228 (38), 227 (98), 185 (42), 183 (40), 158 (27), 155 (23), 149 (41), 91 (22), 89 (47), 77 (31). HRMS (ESI) *m*/*z* calcd for [C_18_H_27_NO_4_ + H]^+^: 322.2013; found: 322.2012.

#### 3.2.2. Step 2: Synthesis and Characterization Data of 2-Benzyl-2-[2-(Dimethylamino)ethyl]malonic Acid (**4**)

A solution of ester **3** (1.16 g, 3.6 mmol) and NaOH (0.504 g, 12.6 mmol) in EtOH (5 mL) was refluxed for 4 h. The resulting suspension was concentrated under reduced pressure, and the residue obtained was dissolved in H_2_O (5 mL). Acetic acid was added dropwise while cooling in an ice water bath (5 °C) until a white solid was obtained. Then, the solid was filtered, washed with cold water (2 × 3 mL) and EtOH (5 mL), and dried in a vacuum oven at 50 °C to yield product **4** as a white-yellow solid at a 75% yield (0.720 g).

*2-Benzyl-2-[2-(dimethylamino)ethyl]malonic acid* (**4**): ^1^H NMR (600 MHz, D_2_O) δ 2.18–2.22 (m, 2 H), 2.87 (s, 6 H), 3.08–3.12 (m, 2 H), 3.18 (s, 2 H), 7.21–7.23 (m, 2 H), 7.33–7.38 (m, 3 H); ^13^C NMR (150 MHz, D_2_O) δ 29.8, 42.7, 43.1, 54.5, 58.5, 127.3, 128.6, 129.6, 136.3, 178.2; FT-IR (film, cm^−1^): 3350, 2920, 2850, 1599, 1494, 1243, 1041, 752. HRMS (ESI) *m*/*z* calcd for [C_14_H_19_NO_4_-H]^−^: 264.1241; found: 264.1248.

#### 3.2.3. Step 3: Synthesis and Characterization Data of 2-[2-(Dimethylamino)ethyl]indan-1-one (**5a**)

Compound **4** (0.72 g, 2.71 mmol) was added to a preheated solution of polyphosphoric acid (0.72 g) in 1 mL of CPME or 2-MeTHF at 90 °C. The resulting brown reaction mixture was then heated under reflux and stirred for 20 min. The reaction was quenched with cold water (1 mL) and basified with an aq. sol. of K_2_CO_3_ (2 M). The mixture was extracted with CPME or 2-MeTHF (3 × 1 mL), washed with H_2_O (5 mL), dried (Na_2_SO_4_), and evaporated to produce **5a** as a yellow oil at a 50% yield (0.280 g) in CPME and a 55% yield (0.303 g) in 2-MeTHF.

*2-[2-(Dimethylamino)ethyl]indan-1-one* (**5a**): ^1^H NMR (600 MHz, CDCl_3_) δ 1.54–1.64 (m, 1 H), 2.10–2.19 (m, 1 H), 2.23 (s, 6 H), 2.40–2.47 (m, 2 H), 2.66–2.73 (m, 1 H), 2.81 (dd, *J* = 17.3 Hz, 4.4 Hz, 1 H), 3.31 (dd, *J* = 17.3, 9.4 Hz, 1 H), 7.28 (d, *J* = 7.4 Hz, 1 H), 7.38 (t, *J* = 7.4 Hz, 1 H), 7.55 (t, *J* = 7.4 Hz, 1 H), 7.72 (d, *J* = 7.4 Hz, 1 H); ^13^C NMR (150 MHz, CDCl_3_) δ 28.3, 32.8, 45.5, 47.3, 57.6, 125.1, 127.3, 128.6, 132.9, 137.4, 147.8, 205.2; FT-IR (film, cm^−1^): 2639, 1699, 1598, 1573, 1471, 1434, 1287, 1220, 1147, 500; GC/MS (70 eV) *m*/*z* (%): 203 (M^+^, 100), 120 (11), 109 (15), 108 (32), 96 (78), 95 (36), 80 (12), 79 (24), 78 (43), 67 (37). HRMS (ESI) *m*/*z* calcd for [C_13_H_17_NO + H]^+^: 204.1383; found: 204.1384.

#### 3.2.4. Step 4: Synthesis and Characterization Data of Dimethyl{2-[5-methyl-3-(1-pyridin-2-ylethyl)-1H-inden-2-yl]ethyl}amine (Dimethindene) (**6**)

To a solution of 2-ethylpyridine (0.326 g, 3.04 mmol, 2.2 equiv) in CPME or 2-MeTHF (2 mL), *n*-BuLi (1.52 mL of a solution in cyclohexanes, 3.04 mmol, 2.2 equiv) was added at RT under nitrogen and stirred for 2 h at this temperature. To the resulting dark red solution, **5a** (0.28 g, 1.38 mmol, 1 equiv) was added dropwise, and the mixture was stirred overnight at RT. The reaction was quenched with cold water, washed with saturated NaHCO_3_ (2 × 2 mL), and extracted with HCl (20%) (2 × 2 mL). The water layer was refluxed for 1 h, cooled to RT, basified with an NH_4_OH sol., and extracted with CPME or 2-MeTHF (3 × 1 mL). The organic phase was dried over anhydrous Na_2_SO_4_. Evaporation of the solvent under reduced pressure afforded the crude product that was purified by flash-chromatography on silica gel (hexane/EtOAc 8:2) to provide the desired product **6** at a 65% yield (0.262 g) both in CPME and in 2-MeTHF.

*Dimethyl{2-[5-methyl-3-(1-pyridin-2-ylethyl)-1H-inden-2-yl]ethyl}amine* (*Dimethindene*) (**6**): ^1^H NMR (600 MHz, CDCl_3_) δ 1.75 (d, *J* = 7.2 Hz, 3 H), 2.26 (s, 6 H), 2.37–2.52 (m, 2 H), 2.61–2.74 (m, 2 H), 3.40 (s, 2 H), 4.48 (q, *J* = 7.2 Hz, 1 H), 6.99–7.11 (m, 4 H), 7.16–7.18 (m, 1 H), 7.35–7.37 (m, 1 H), 7.47–7.51 (m, 1 H), 8.60 (d, *J* = 4.8 Hz, 1 H); ^13^C NMR (150 MHz, CDCl_3_) δ 20.9, 29.2, 39.9, 40.6, 45.4, 59.5, 120.7, 121.7, 122.9, 124.1, 128.2, 137.5, 141.4, 142.6, 143.6, 148.2, 164.7; FT-IR (film, cm^−1^): 2920, 2750, 1580, 1494, 1253, 1041, 742; GC/MS (70 eV) *m*/*z* (%): 292 (M^+^, 7), 168 (68), 119 (25), 108 (20), 107 (17), 95 (38), 94 (33), 77 (62), 43 (11). HRMS (ESI) *m*/*z* calcd for [C_20_H_24_O_2_ + H]^+^: 293.2012; found: 293.2013.

### 3.3. Current Industrial Synthesis of Dimethindene (**6**) in VOCs (Toluene, Et_2_O)

#### 3.3.1. Step 1: Synthesis of 2-Benzyl-2-[2-(Dimethylamino)ethyl]malonic Acid Diethyl ester (**3**)

To a refluxing suspension of NaH (0.096 g, 4 mmol, 1 equiv) in toluene (20 mL) 2-benzylmalonic acid diethyl ester **2** (1.0 g, 4 mmol, 1 equiv) was added dropwise. The reaction mixture was refluxed for 1 h to produce a clear yellow solution. 2-chloro-*N,N*-dimethylethan-1-amine (0.86 g, 8 mmol, 2 equiv) was then added dropwise, and the reaction mixture was refluxed for 6 h. The resulting suspension was extracted with HCl (5%) (3 × 1 mL). The aqueous layers were combined, basified with NH_4_OH (pH = 11–12), and extracted with Et_2_O (3 × 4 mL). The combined organic layers were dried (Na_2_SO_4_) and concentrated to yield compound **3** as a yellow oil at a 63% yield (0.819 g).

#### 3.3.2. Step 2: Synthesis of 2-Benzyl-2-[2-(Dimethylamino)ethyl]malonic Acid (**4**)

As described in Section 3.2.2.

#### 3.3.3. Step 3: Synthesis of 2-[2-(Dimethylamino)ethyl]indan-1-one (**5a**)

Compound **4** (0.72 g, 2.71 mmol) was added to polyphosphoric acid (1.15 g) at 110 °C with overhead stirring. The resulting brown reaction mixture was heated to 140–150 °C and stirred for 20 min. The reaction was finally quenched with the cautious addition of ice chips and then with cold water (1 mL), and it was basified with an aq. sol. of K_2_CO_3_ (2 M). The mixture was extracted with Et_2_O (3 × 2 mL), washed with water (5 mL), dried (Na_2_SO_4_), and evaporated under reduced pressure to yield **5a** as a yellow oil at a 20% yield (0.110 g).

#### 3.3.4. Step 4: Synthesis of Dimethyl{2-[5-Methyl-3-(1-pyridin-2-ylethyl)-1H-inden-2-yl]ethyl}amine (**6**)

To a solution of 2-ethylpyridine (0.326 g, 3.04 mmol, 2.2 equiv) in anhydrous Et_2_O (3 mL), *n*-BuLi (1.52 mL of a solution in cyclohexanes, 3.04 mmol, 2.2 equiv) was added at −78 °C under nitrogen and stirred for 2 h at this temperature. To the resulting dark red solution, **5a** (0.28 g, 1.38 mmol, 1 equiv) was added dropwise, and the resulting mixture was stirred overnight at RT. The reaction was quenched with cold water, washed with saturated NaHCO_3_ (2 × 2 mL), and extracted with HCl (20%) (2 × 2 mL). The water layer was refluxed for 1 h, cooled to RT, basified with an NH_4_OH sol., and extracted with Et_2_O (3 × 1 mL). The organic phase was dried over anhydrous Na_2_SO_4_. Evaporation of the solvent under reduced pressure afforded the crude product that was purified by flash-chromatography on silica gel (hexane/EtOAc 8:2) to provide dimethindene (**6**) at a 60% yield (0.242 g).

## 4. Conclusions

An environmentally sustainable procedure was developed for the synthesis of the antihistamine drug dimethindene (**6**) using green solvents 2-MeTHF and CPME belonging to classes 3 and 2, respectively, in agreement with the principles of green chemistry and following the recommendations of the ACS GCI Pharmaceutical Roundtable to redesign the chemical manufacturing of drugs by substituting the use of hazardous chemicals and fossil fuels and/or by reducing the production of waste to a minimum. The old synthetic industrial procedure, consisting of four steps and based on VOCs and harsh reaction conditions, has been now revisited and optimized by employing stoichiometric amounts of reagents and milder conditions, and the outcome of each step was compared when alternatively using 2-MeTHF or CPME as the solvent in place of VOCs. As a result, the overall yield of the process improved from 10% (with VOCs) to 21–22% (with 2-MeTHF or CPME) with a simplified work-up procedure. The decreased environmental impact of the newly reported approach is supported by the calculation of Quantitative Metrics of the CHEM21 Metrics Toolkit. Thus, an old and hazardous industrial process for the synthesis of a drug was usefully transformed into a more environmentally friendly synthetic methodology.

## Figures and Tables

**Table 1:** Quantitative metrics for each step ^a^ of the classical synthesis of dimethindene (**6**) in VOCs or in CPME or in 2-MeTHF.

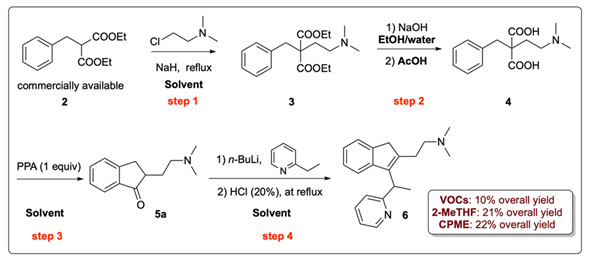											
	Reaction Solvent	Yield	AE (%)	RME (%)	OE (%)	EM (%)	MI ^b^ (g g^−1^)	MI_WU_ ^c^ (g g^−1^)	RI ^d^	RP (%)	E-Factor ^e^
**Step 1**	VOCs	63%	84.2	41.9	49.8	4.2	23.6	41.6	7.3	17.5	33.1
	CPME	90%	84.2	75.8	90.0	75.8	8.8	18.4	17.1	92.9	12.2
	2-MeTHF	80%	84.2	67.4	80.0	67.4	9.9	20.7	19.2	92.8	13.8
**Step 2**	/	75%	73.4	43.4	59.1	19.1	10.8	24.7	22.4	90.6	9.8
**Step 3**	VOCs	20%	55.7	5.9	17.2	1.8	17.0	110.4	54.5	49.4	54.9
	CPME	50%	55.7	19.4	34.8	19.4	8.2	38.8	33.7	86.9	16.4
	2-MeTHF	55%	55.7	21.0	37.7	21.0	7.6	35.9	31.2	87.0	15.1
**Step 4**	VOCs	60%	78.1	13.8	17.5	4.0	16.2	58.5	33.1	56.6	24.1
	CPME	65%	78.1	15.0	19.2	14.9	13.3	54.0	46.9	86.9	22.1
	2-MeTHF	65%	78.1	15.0	19.2	14.9	13.3	54.0	46.9	86.9	22.1

^a^ In order to have comparable data, the reactions of each step in VOCs (toluene and Et_2_O), CPME, or 2-MeTHF were carried out with the same quantity of reagents. ^b^ Mass intensity (MI): chemicals and reaction solvents. ^c^ Mass intensity (MI)_WU_: chemicals and reaction solvents, solvents, and reagents in workup. ^d^ Renewable sources: water, CPME, 2-MeTHF, acetic acid, and EtOH. ^e^ This value does not consider the amount of water and water solution used for workup.

**Table 2:** Quantitative metrics for the process of synthesis of dimethindene (**6**) in VOCs or in CPME or in 2-MeTHF.

Reaction Solvent	Yield	AE (%)	RME (%)	OE (%)	EM (%)	PMI_RXN_^a^ (g g^−1^)	PMI_WU_^b^ (g g^−1^)	RI ^c^	RP (%)	E-Factor ^d^
VOCs	10%	42.2	3.3	7.8	0.7	136.2	322.0	149.4	46.4	188.0
CPME	22%	42.2	4.1	9.3	3.1	90.4	244.4	220.1	90.1	121.6
2-MeTHF	21%	42.2	3.9	8.8	3.0	94.0	254.1	228.8	90.0	126.5

^a^ Process mass intensity (PMI)_RXN_: chemicals and reaction solvents. ^b^ Process mass intensity (PMI)_WU_: chemicals and reaction solvents, solvents, and reagents in workup. ^c^ Renewable sources: water, CPME, 2-MeTHF, acetic acid, and EtOH. ^d^ This value does not consider the amount of water solution used for workup.

## Data Availability

Not applicable.

## Supplementary Materials

The following supporting information can be downloaded at: https://www.mdpi.com/article/10.3390/molecules27217594/s1, typical metrics applied at Zero, First, and Second pass according to the CHEM21 Metrics Toolkit; quantitative metrics of classical and eco-friendly approach for the synthesis of Dimethindene (**6**); E-factor calculation for the synthesis of Dimethindene (**6**); Tables S1–S4: various experimental procedures; ^1^H and ^13^C NMR spectra of compounds **3**, **4**, **5a**, and **6**.

## Sample Availability

Not available.
