# Supplementary material for: Green Solvents for Eco-Friendly Synthesis of Dimethindene: A Forward-Looking Approach
Source: Molecules. 2022 Nov 5;27(21):7594. doi: 10.3390/molecules27217594 (PMC9654048; doi:10.3390/molecules27217594)
Supplement: Supplementary file 1 [file molecules-27-07594-s001.zip › molecules-1988519-supplementary.pdf]

Supplementary Materials

Green Solvents for Eco-Friendly Synthesis of Dimethindene: A Forward-Looking Approach

Table of Contents

1.	Typical metrics applied at Zero, First and Second Pass according to the CHEM21 Metrics Toolkit.	S2
2.	Quantitative metrics of classical and eco-friendly approach for the synthesis of Dimethindene (**6**).	S3
3.	E-factor calculation for the synthesis of Dimethindene (**6**).	S14
4.	Experimental Procedures. α-Functionalization of indanone (**7**).	S18
4.1.	Table S1. Treatment of 1-indanone (**7**) with different bases and reaction with electrophiles (Scheme 3,a-c, main text).	S18
4.2.	Table S2. Ru-catalyzed hydrogen-borrowing C–C bond formation: reaction between 1-indanone (**7**) and 2-(dimethylamino)ethan-1-ol (**14e**) (Scheme 3,e, main text).	S19
4.3.	Table S3. α-Functionalization of 1-indanone (**7**) via enamine **12** (Scheme 3,e, main text).	S20
4.4.	Table S4. Reaction between 2-chloro-*N*,*N*-dimethylethan-1-amine (**14a**) and β-ketoester **13** (Scheme 3,f, main text).	S21
5.	^{1}H and ^{13}C NMR spectra of compounds **3**, **4**, **5a**, and **6**.	S22

1. Typical metrics applied at Zero, First and Second Pass according to the CHEM21 Metrics Toolkit.[1]

Metric	Acronym	Formula
**Percentage yield**	Y (%)	$\frac{\text{mol of product}}{\text{mol of limiting reactant}} \times 100$
**Percentage conversion**	Conv. (%)	$\frac{\text{final mass of limiting reactant (kg)}}{\text{initial mass of limiting reactant (kg)}} \times 100$
**Percentage selectivity**	Sel. (%)	$\frac{\text{\% Yield}}{\text{\% Conv.}} \times 100$
**Reaction mass efficiency**	RME	$\frac{\text{mass of product (kg)}}{\sum \text{mass reagents (kg)}} \times 100$
**Atom economy**	AE	$\text{AE} = \frac{\text{m. w product}}{\sum \text{m. w. reagents}} \times 100$
**Effective mass yield**	EM	$\frac{\text{mass of products (kg)}}{\text{mass of non benign reagents (kg)}} \times 100$
**Optimum efficiency**	OE	$\frac{\text{RME}}{\text{AE}} \times 100$
**Mass intensity**	MI	$\frac{\text{total mass used in a process step (kg)}}{\text{mass of product (kg)}}$
**Process mass intensity**	PMI	$\frac{\text{total mass in a process}}{\text{mass of product}}$
**Renewables intensity**	RI	$\frac{\text{mass of all renewably derivable materials used}}{\text{mass of product}}$
**Renewables percentage**	RP	$\frac{\text{RI}}{\text{PMI}} \times 100$
**Detailed solvents**	*Recommended, Problematic, Hazardous, Highly Hazardous*	
**Energy**	• 0–70 °C *Green Flag*<br>• To 140 °C *Yellow Flag*<br>• Reflux *Red Flag*	
**Workup**	• *Green Flag*: quenching, filtration, centrifugation, crystallisation,<br>• *Amber Flag*: solvent exchange, quenching into aqueous solvent.<br>• *Red Flag*: chromatography, high temperature distillation	

[1] C. R. McElroy, A. Constantinou, L. C. Jones, L. Summerton, J. H. Clark, *Green Chem*. **2015**, *17*, 3111.

2. Quantitative metrics of classical and eco-friendly approach for the synthesis of Dimethindene (**6**).

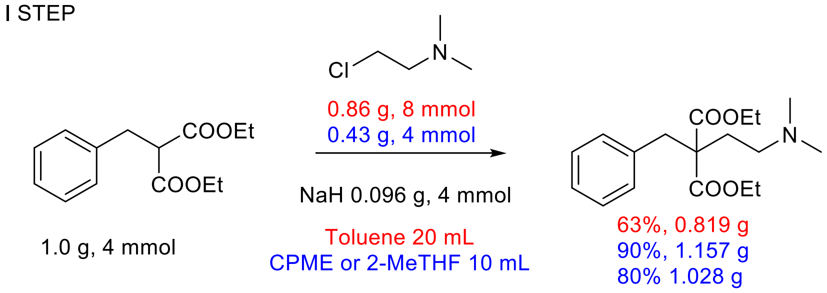

Work-up
The resulting suspension was extracted with HCl (5%) (3 × 1 mL). The aqueous layers were combined, basified with NH_4OH (pH = 11-12), and extracted with CPME (3×2 mL, 0.86 g/mL, 5.16 g) or Et_2O (3×4 mL, 0.713 g/mL, 8.56). The combined organic layers were dried (Na_2SO_4) and concentrated to give a yellow oil

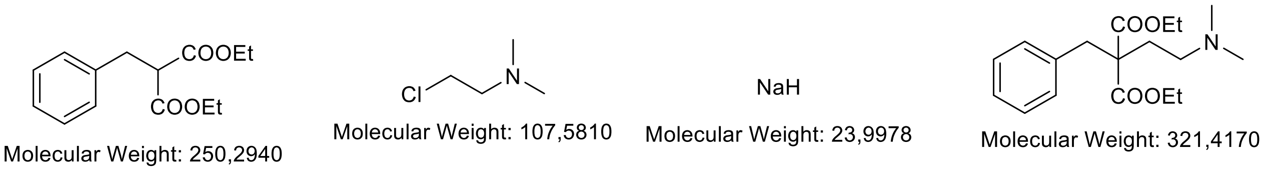

Workup: Amber Flag (solvent exchange, quenching into aqueous solvent).

Detailed Solvent: Toluene and Et_2O: problematic and highly hazardous.

Detailed Solvent: CPME or 2-MeTHF: recommended

AE

M. W. reactants 250.3 + 107.6 + 24 = 381.9 AE = 321.4/381.9 % = 84.2%

RME

Classical Industrial Synthesis 0.819/1.956 % = 41.9%

Reaction in CPME 1.157/1.526 % = 75.8%

Reaction in 2-MeTHF 1.028/1.526 % = 67.4%

OE

Classical Industrial Synthesis 41.9/84.2 % = 49.8%

Reaction in CPME 75.8/84.2 % = 90.0%

Reaction in 2-MeTHF 67.4/84.2 % = 80.0%

EM

Classical Industrial Synthesis

Mass of non benign reagents 1.0 g + 0.86 g (2-chloro-*N*,*N*-dimethylethan-1-amine) + 0.096 (NaH) + 17.4 g (toluene) = 19.356

EM = 0.819/19.356 % = 4.2%

Reaction in CPME

Mass of non benign reagents 1.0 g + 0.43 g (2-chloro-*N*,*N*-dimethylethan-1-amine) + 0.096 (NaH) = 1.526 g

EM = 1.157/1.526 % = 75.8%

Reaction in 2-MeTHF

Mass of non benign reagents 1.0 g + 0.43 g (2-chloro-*N*,*N*-dimethylethan-1-amine) + 0.096 (NaH) = 1.526 g

EM = 1.028/1.526 % = 67.4%

MI

Classical Industrial Synthesis

Total amount of reactants: 1.0 g + 0.86 g + 0.096 g + 17.4 g (toluene) + [*8.56 g (Et_2O) + 6.0 g (water)*] = 19.36 g [33.92 g]

Amount of the final product: 0.819 g

MI 19.36/0.819 = 23.6

MI_{WU} 33.92/0.819 = 41.6

Reaction in CPME

Total amount of reactants: 1.0 g + 0.43 g + 0.096 g + 8.6 g (CPME) + [*5.16 g (CPME) + 6.0 g (water)*] = 10.13 g [21.29 g]

Amount of the final product: 1.157 g

MI 10.13/1.157 = 8.8

MI_{WU} 21.29/1.157 = 18.4

Reaction in 2-MeTHF

Total amount of reactants: 1.0 g + 0.43 g + 0.096 g + 8.6 g (2-MeTHF) + [*5.16 g (2-MeTHF) + 6.0 g (water)*] = 10.13 g [21.29 g]

Amount of the final product: 1.028 g

MI 10.13/1.028 = 9.9

MI 21.29/1.028 = 20.7

Ri and RP

Reaction in toluene

Renewable sources: 6.0 g (water)

RI: 6.0/ 0.819 = 7.3   RP: 7.3/ 41.6 % = 17.5%

Reaction in CPME

Renewable sources: 6.0 g (water) + 13.76 g (CPME) = 19.76 g

RI: 19.76 / 1.157 = **17.1** **RP**: 17.1 / 18.4 % = **92.9%**

Reaction in 2-MeTHF

Renewable sources: 6.0 g (water) + 13.76 (2-MeTHF) = 19.76 g

RI: 19.76 / 1.028 = **19.2** **RP**: 19.2 / 20.7 % = **92.8%**

II STEP

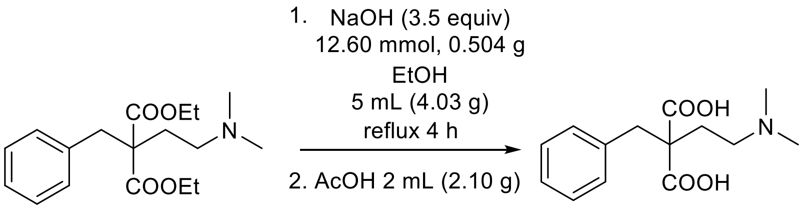

1.157 g, 3.60 mmol

75%, 0.720g

Work-up
The solid product was filtered, washed with cold water (2×3 mL) and ethanol (5 mL), and dried in a vacuum oven at 50 °C to give a white-yellow solid

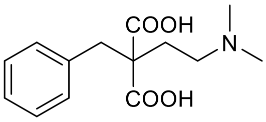

Molecular Weight: 265,3090

NaOH

Molecular Weight: 39,9968

Workup: Green Flag (filtration).

Detailed Solvent: EtOH, water: recommended

AE

M. W. reactants 321.4 + 40 = 361.4 AE = 265.3/361.4 % = 73.4%

RME

0.72/1.66 % = 43.4%

OE

43.44/73.4 % = 59.1 %

EM

0.72 g/ (1.16 g + 0.504 g + 2.10 g = 3.76) % = 19.1%

MI

Total amount of reactants: 1.16 g + 0.504 g + 4.03 g + 2.10 g + [*4.03 g (EtOH) +6.0 g (water)*] = 7.79 g [17.82 g]

Amount of the final product: 0.72 g

MI = 7.79/ 0.72 = 10.82

MI_{WU} = 17.82/ 0.72 g = 24.75

RI and RP II STEP

Renewable sources: 8.06 g (EtOH) + 2.1 g (AcOH) + 6.0 g (water) = 16.16 g

RI: 16.16/ 0.72 = 22.4 RP: 22.4 / 24.75 % = 90.6%

III STEP

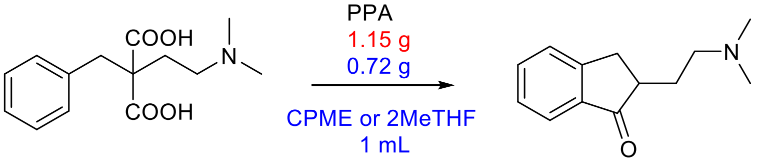

0.72 g, 2.71 mmol

VOCs: 20%, 0.11 g
CPME: 50%, 0.28 g
2-MeTHF: 55%. 0.303 g

Work-up
The reaction was quenched with cold water (1 mL) and basified with K_2CO_3 (2 M). The mixture was extracted with CPME (3×1 mL, 2.58 g), or Et_2O (3×2 mL, 4.28 g) washed with water (5 mL), dried (Na_2SO_4), and evaporated to give a yellow oil in yield

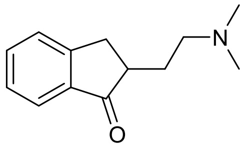

Molecular Weight: 203,2850

Workup: Amber Flag (solvent exchange, quenching into aqueous solvent).

Detailed Solvent: CPME or 2-MeTHF: recommended

AE

203.3/ (265.3 + 100) % = 55.7%

RME

Classical Industrial Synthesis

Amount of the final product: 0.11 g

Total amount of reactants: 0.72 g + 1.15 g = 1.87 g

RME: 0.11/1.87 % = 5.9%

Reaction in CPME

Amount of the final product: 0.28 g

Total amount of reactants: 0.72 g + 0.72 g = 1.44 g

RME = 0.28/1.44 %= 19.4%

Reaction in 2-MeTHF

Amount of the final product: 0.303 g

Total amount of reactants: 0.72 g + 0.72 g = 1.44 g

RME = 0.303/1.44 %= 21.0%

OE

Classical Industrial Synthesis 9.6/55.7 % = 17.2%

Reaction in CPME 19.4/55.7 % = 34.8%

Reaction in 2-MeTHF 21.0/ 55.7 % = 37.7%

EM

Classical Industrial Synthesis

Amount of the final product: 0.11 g

Total mass of non benign reagents: 0.72 g + 1.15 g + 4.28 g (Et_2O) = 6.15 g

EM = 0.11 / 6.15 % = 1.8%

Reaction in CPME

Amount of the final product: 0.28 g

Total mass of non benign reagents: 0.72 g + 0.72 g = 1.44 g

EM: 0.28/1.44 = 19.4%

Reaction in 2-MeTHF

Amount of the final product: 0.303 g

Total mass of non benign reagents: 0.72 g + 0.72 g = 1.44 g

EM: 0.303/1.44 = 21.0%

MI

Classical Industrial Synthesis

Total amount of reactants: 0.72 g + 1.15 g + [*4.28 g (Et_2O) + 6 g (water)*] = 1.87 g [12.15 g]

Amount of the final product: 0.11 g

MI: 1.87/0.11 = 17.0

MI_{WU}: 12.15/ 0.11 = 110.4

Reaction in CPME

Total amount of reactants: 0.72 g + 0.72 g + 0.86 g (CPME) + [*2.58 g (CPME) + 6 g (water)*] = 2.30 g [10.88 g]

Amount of the final product: 0.28 g

MI: 2.3/ 0.28 = 8.2

MI_{WU}: 10.88/0.28 = 38.8

Reaction in 2-MeTHF

Total amount of reactants: 0.72 g + 0.72 g + 0.86 g (2-MeTHF) + [*2.58 g (2-MeTHF) + 6 g (water)*] = 2.30 g [10.88 g]

Amount of the final product: 0.303 g

MI: 2.3/0.303 = 7.6

MI_{WU}: 10.88/0.303 = 35.9

RI and RP

Reaction in toluene Renewable sources: 6.0 g (water)

RI: 6.0/ 0.11 = 54.5 **RP**: 54.5/ 110.4% = 49.4%

Reaction in CPME Renewable sources: 6.0 g (water) + 3.44 g (CPME) = 9.44 g

RI: 9.44/ 0.28 = 33.7 **RP**: 33.7/ 38.8 % = 86.9%

Reaction in 2-MeTHF Renewable sources: 6.0 g (water) + 3.44 g (2-MeTHF) = 9.44 g

RI: 9.44 / 0.303 = 31.2 **RP**: 31.2 / 35.9% = 87.0%

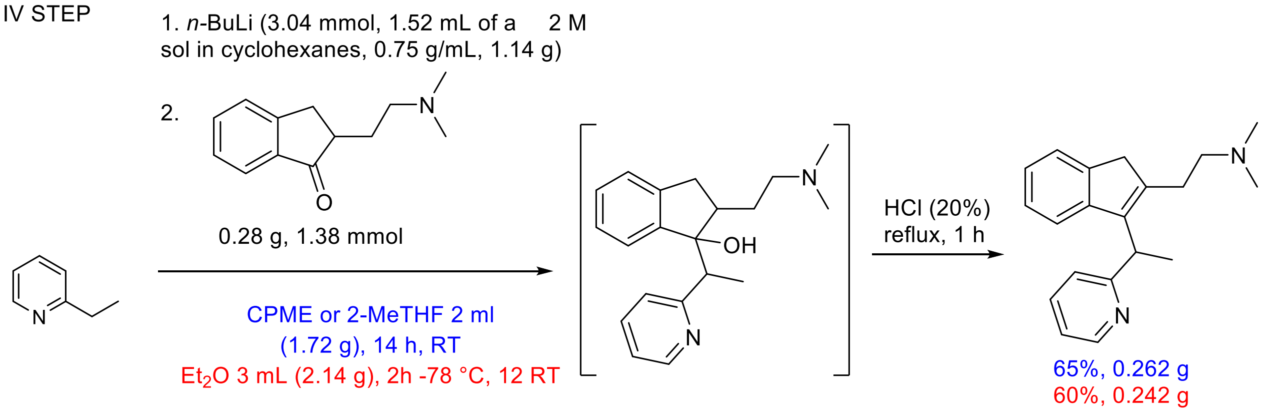

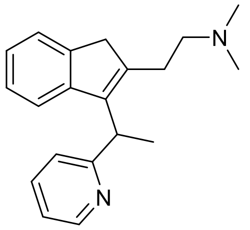

Molecular Weight: 292,4260

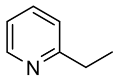

Molecular Weight: 107,1560

Work-up

The reaction was quenched with cold water, washed with saturated $NaHCO_3$ (2x2 mL) and extracted with HCl (20%) (2x2 mL). The water layer was refluxed for 1 h, cooled to RT, basified with NH_4OH sol. and extracted with CPME (3×1 mL) or Et_2O (3×1 mL). The organic phase was dried over anhydrous Na_2SO_4. Evaporation of the solvent under reduced pressure afforded the crude that was purified by flash-chromatography on silica gel

Workup: Amber Flag (solvent exchange, quenching into aqueous solvent) / Red Flag (chromatography).

Detailed Solvent: Et_2O: problematic.

Detailed Solvent: CPME or 2-MeTHF: recommended

Energy: Red Flag, -78 °C

Energy: Green Flag, 0 °C

AE

292.4/ (203.3 + 107.2 + 64.1) % = 78.1%

RME

Classical Industrial Synthesis

Amount of the final product: 0.242 g

Total amount of reactants: 0.326 g + 1.14 g + 0.28 g = 1.75 g

RME: 0.242/1.75 % = 13.8 %

Reaction in CPME

Amount of the final product: 0.26 g

Total amount of reactants: 0.326 g + 1.14 g + 0.28 g = 1.75 g

RME = 0.262/1.75 %= 15.0%

Reaction in 2-MeTHF

Amount of the final product: 0.262 g

Total amount of reactants: 0.326 g + 1.14 g + 0.28 g = 1.75 g

RME = 0.262/1.75 %= 15.0%

OE

Classical Industrial Synthesis 13.8/78.1 % = 17.7 %

Reaction in CPME 15/78.1 % = 19.2 %

Reaction in 2-MeTHF 15/78.1 % = 19.2 %

EM

Classical Industrial Synthesis

Amount of the final product: 0.24 g

Total mass of non benign reagents: 0.326 g + 1.14 g + 0.28 g + 2.14 g (Et_2O) + 2.14 g (Et_2O) = 6.03 g

EM = 0.24 / 6.03 % = 4.0 %

Reaction in CPME

Amount of the final product: 0.26 g

Total mass of non benign reagents: 0.326 g + 1.14 g + 0.28 g = 1.75 g

EM: 0.26/1.75 = 14.9 %

Reaction in 2-MeTHF

Amount of the final product: 0.26 g

Total mass of non benign reagents: 0.326 g + 1.14 g + 0.28 g = 1.75 g

EM: 0.26/1.75 = 14.9 %

MI

Classical Industrial Synthesis

Total amount of reactants: 0.326 g + 1.14 g + 0.28 g + 2.14 g (Et_2O) + *[2.14 g (Et_2O) + 8.0 g (water)]* = 3.89 g [14.03 g]

Amount of the final product: 0.24 g

MI: 3.89/0.24 = 16.2

MI_{WU} = 14.03/0.24 = 58.5

Reaction in CPME

Total amount of reactants: 0.326 g + 1.14 g + 0.28 g + 1.72 g (CPME) + [*2.58 g (CPME) + 8.0 g (water)]* = 3.46 g [14.04 g]

Amount of the final product: 0.26 g

MI: 3.46/0.26 = 13.3

MI_{WU} = 14.04/0.26 = 54.0

Reaction in 2-MeTHF

Total amount of reactants: 0.326 g + 1.14 g + 0.28 g + 1.72 g (2-MeTHF) + [*2.58 g (2-MeTHF) + 8.0 g (water)]* = 3.46 g [14.04 g]

Amount of the final product: 0.26 g

MI: 3.46/0.26 = 13.3

MI_{WU} = 14.04/0.26 = 54.0

RI and RP IV STEP

Reaction in toluene Renewable sources: 8.0 g (water)

RI: 8.0/ 0.242 = 33.1 RP: 33.1/ 58.5 % = 56.6 %

Reaction in CPME Renewable sources: 8.0 g (water) + 4.30 (CPME) = 12.30 g

RI: 12.3/ 0.262 = 46.9 RP: 46.9/ 54.0 % = 86.9 %

Reaction in 2-MeTHF Renewable sources: 8.0 g (water) + 4.30 (2-MeTHF) = 12.30 g

RI: 12.3 / 0.262 = 46.9 RP: 46.9/ 54.0 % = 86.9%

Process for Dimethindene Synthesis

AE 292.4/ [(250.3 + 107.6 + 24.0) + 40.0 + 100 + (107.2 + 64.1)] % = 42.2 %

RME

Classical Industrial Synthesis 0.242/ (1.956 + 1.66 + 1.87 +1.75 = 7.236) % = 3.3 %

Reaction in CPME 0.262/ (1.526 + 1.66 + 1.44 + 1.75 = 6.376) % = 4.1 %

Reaction in 2-MeTHF 0.246/ (1.526 + 1.66 + 1.44 +1.75) % = 3.9 %

OE

Classical Industrial Synthesis 3.3/42.2 % = 7.8 %

Reaction in CPME 4.1/42.2 % = 9.3 %

Reaction in 2-MeTHF 3.9/42.2 % = 8.8 %

PMI

Classical Industrial Synthesis

Total amount of reactants: 19.4 + 7.79 + 1.87 + 3.89= 32.95

Amount of the final product: 0.242 g

PMI 32.95/0.242 = 136.2

Reaction in CPME

Total amount of reactants: 10.13 + 7.79 + 2.3 + 3.46= 23.68

Amount of the final product: 0.262 g

PMI 23.68/ 0.262 = 90.4

Reaction in 2-MeTHF

Total amount of reactants: 10.13 + 7.79 + 2.3 + 3.46= 23.68

Amount of the final product: 0.252 g

PMI 23.68/ 0.252 = 94.0

PMI_{WU}

Classical Industrial Synthesis

Total amount of reactants: 33.92 + 17.82 + 12.15 + 14.03= 77.92

Amount of the final product: 0.242 g

PMI 77.92/0.242 = 322.0

Reaction in CPME

Total amount of reactants: 21.39 + 17.82 + 10.88 + 14.04= 64.03

Amount of the final product: 0.262 g

PMI 64.03/ 0.262 = 244.4

Reaction in 2-MeTHF

Total amount of reactants: 21.39 + 17.82 + 10.88 + 14.04= 64.03

Amount of the final product: 0.252 g

PMI 64.03/ 0.252 = 254.1

EM

Classical Industrial Synthesis

Total mass of non benign reagents: 19.36 g + 3.76 g + 6.15 g + 6.03 g = 35.30 g

Amount of the final product: 0.242 g

EM 0.242/35.30 % = 0.7%

Reaction in CPME

Total mass of non benign reagents: 1.526 g + 3.76 g +1.44 g + 1.75 g = 8.32

Amount of the final product: 0.262 g

EM 0.262/8.32 % = 3.1%

Reaction in 2-MeTHF

Total mass of non benign reagents: 1.526 g + 3.76 g +1.44 g + 1.75 g = 8.32

Amount of the final product: 0.246 g

EM 0.246/8.32 % = 3.0%

RI and RP

Classical Industrial Synthesis

RI: (6.0 + 16.16 + 6.0 + 8.0) g / 0.242 g = 149.4

RP 149.4/322.0 =46.4%

Reaction in 2-MeTHF

RI (19.76 + 16.16 + 9.44 + 12.3)/0.252 = 228.8

RP 228.8 / 254.1 %= 90.0%

Reaction in CPME

RI (19.76 + 16.16 + 9.44 + 12.3)/0.262 = 220.1

RP 220.1 / 244.4 %= 90.1%

3. E-factor calculation for the synthesis of Dimethindene (6).

According to its original definition (*Green Chem*. **2007**, *9*, 1273), the Sheldon E-factor value (total mass of waste/mass of product) takes into account only the mass of waste generated in a process, and its calculation is performed by simply dividing the sum of the molecular weight of all substances produced by molecular weight of the desired products, with reference to the stoichiometric equation. We have followed this general equation in our own calculation.

I STEP

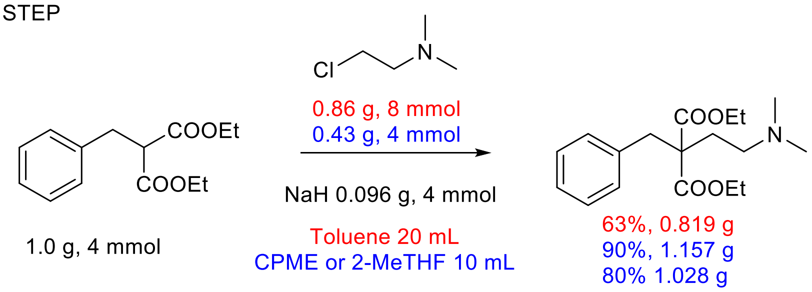

Work-up
The resulting suspension was extracted with HCl (5%) (3 × 1 mL). The aqueous layers were combined, basified with NH_4OH (pH = 11-12), and extracted with CPME (3×2 mL, 0.86 g/mL, 5.16 g) or Et_2O (3×4 mL, 0.713 g/mL, 8.56). The combined organic layers were dried (Na_2SO_4) and concentrated to give a yellow oil

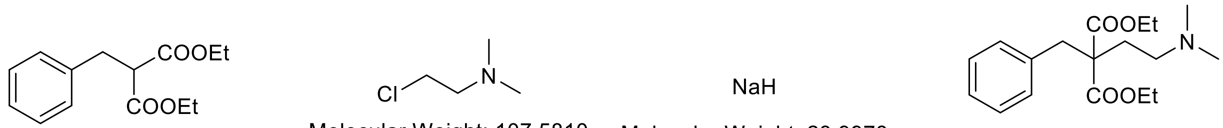

Molecular Weight: 250,2940 | Molecular Weight: 107,5810 | Molecular Weight: 23,9978 | Molecular Weight: 321,4170

Reaction in toluene

Total amount of reactants: 1.0 g + 0.86 g + 0.096 g + 17.4 g (toluene) + 8.56 g (Et_2O) = 27.92 g

Amount of the final product: 0.819 g

Amount of waste: 27.92 g – 0.819 g = 27.10 g

E-factor = amount of waste/amount of product = 27.10/0.819 g = **33.1**

Reaction in CPME

Total amount of reactants: 1.0 g + 0.43 g + 0.096 g + 8.6 g (CPME) + 5.16 g (CPME) = 15.29 g

Amount of the final product: 1.157 g

Amount of waste: 15.29 g – 1.157 g = 14.133 g

E-factor = amount of waste/amount of product = 14.13/1.157 g = **12.2**

Reaction in 2-MeTHF

Total amount of reactants: 1.0g + 0.43g + 0.096g + 8.6 g (2-MeTHF) + 5.16 g (2-MeTHF) = 15.29 g

Amount of the final product: 1.028 g

Amount of waste: 15.29 g – 1.028 g = 14.262 g

E-factor = amount of waste/amount of product = 14.262/1.028 g = **13.8**

II STEP

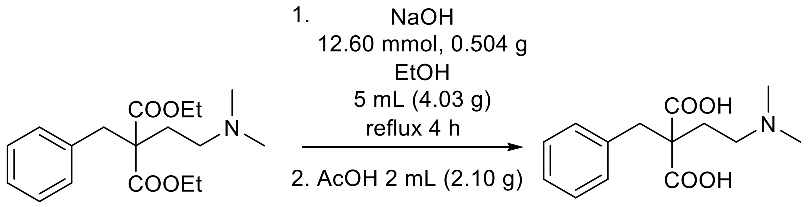

1.16 g, 3.60 mmol

75%, 0.72 g

Work-up
The solid product was filtered, washed with cold water (2×3 mL) and ethanol (5 mL), and dried in a vacuum oven at 50 °C to give a white-yellow solid

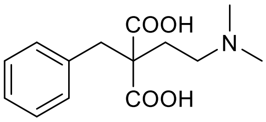

Molecular Weight: 265,3090

Total amount of reactants: 1.16 g + 0.504 g + 4.03 g + 2.10 g = 7.79 g

Amount of the final product: 0.72 g

Amount of waste: 7.79 g – 0.72 g = 7.07 g

E-factor = amount of waste/amount of product = 7.07/0.72 g = **9.8**

III STEP

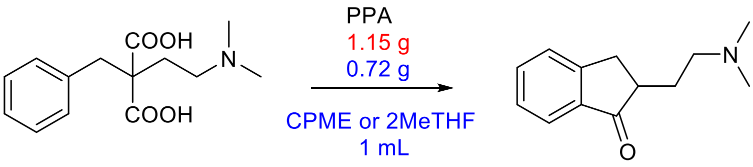

0.72 g, 2.71 mmol

20%, 0.11 g
50%, 0.28 g
55%. 0.303 g

Work-up
The reaction was quenched with cold water (1 mL) and basified with K_2CO_3 (2 M). The mixture was extracted with CPME (3×1 mL, 2.58 g), or Et_2O (3×2 mL, 4.28 g) washed with water (5 mL), dried (Na_2SO_4), and evaporated to give a yellow oil in yield

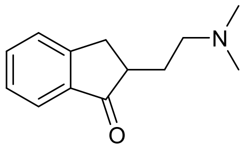

Molecular Weight: 203,2850

Classical Industrial Synthesis

Total amount of reactants: 0.72 g + 1.15 g + 4.28 g (Et_2O) = 6.15 g

Amount of the final product: 0.11 g

Amount of waste: 6.15 g – 0.11 g = 6.04 g

E-factor = amount of waste/amount of product = 6.04/0.11 g = **54.9**

Reaction in CPME

Total amount of reactants: 0.72 g + 0.72 g + 0.86 g (CPME) + 2.58 g (CPME) = 4.88 g

Amount of the final product: 0.28 g

Amount of waste: 4.88 g – 0.28 g = 4.60 g

E-factor = amount of waste/amount of product = 4.60/0.28 g = **16.4**

Reaction in 2-MeTHF

Total amount of reactants: 0.72 g + 0.72 g + 0.86 g (2-MeTHF) + 2.58 g (2-MeTHF) = 4.88 g

Amount of the final product: (55%) 0.303 g

Amount of waste: 4.88 g – 0.303 g = 4.577 g

E-factor = amount of waste/amount of product = 4.577/0.303 g = **15.1**

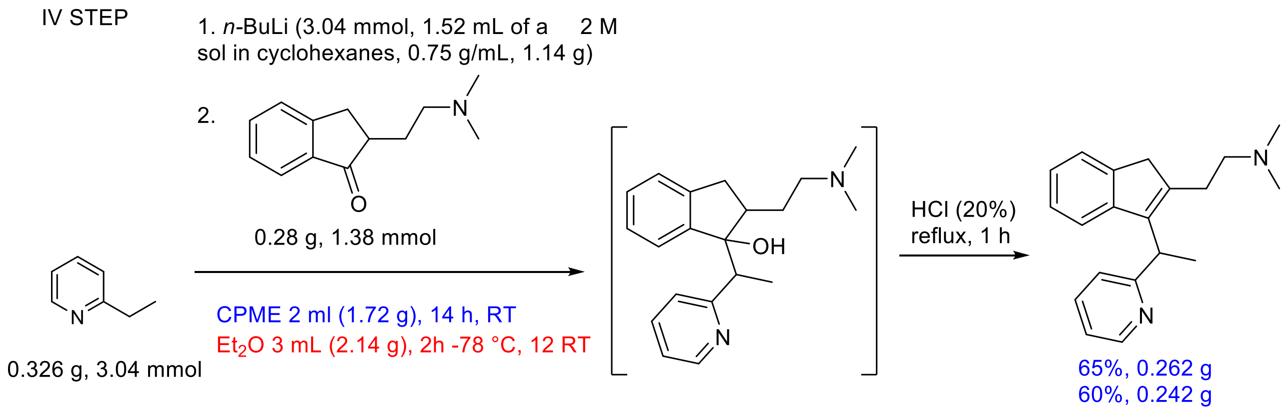

Work-up

The reaction was quenched with cold water (2 mL), washed with saturated $NaHCO_3$ (2x2 mL) and extracted with HCl (20%) (2x2 mL). The water layer was refluxed for 1 h, cooled to RT, basified with NH_4OH sol. and extracted with CPME (3×1 mL) or Et_2O (3×1 mL). The organic phase was dried over anhydrous Na_2SO_4. Evaporation of the solvent under reduced pressure afforded the crude that was purified by flash-chromatography on silica gel

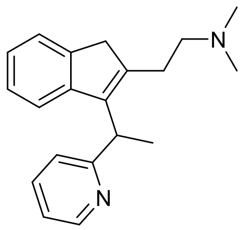

Molecular Weight: 292,4260

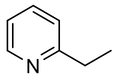

Molecular Weight: 107,1560

Classical Industrial Synthesis

Total amount of reactants: 0.326 g + 1.14 g + 0.28 g + 2.14 g (Et_2O) + 2.14 g (Et_2O) = 6.03 g

Amount of the final product: 0.24 g

Amount of waste: 6.03 g – 0.24 g = 5.79 g

E-factor = amount of waste/amount of product = 5.79/0.24 g = **24.1**

Reaction in CPME

Total amount of reactants: 0.326 g + 1.14 g + 0.28 g + 1.72 g (CPME) + 2.58 g (CPME) = 6.04 g

Amount of the final product: 0.26 g

Amount of waste: 6.04 g – 0.26 g = 5.78 g

E-factor = amount of waste/amount of product = 5.78/0.26 g = **22.1**

Reaction in 2-MeTHF

Total amount of reactants: 0.326 g + 1.14 g + 0.28g + 1.72g (2-MeTHF) + 2.58g (2-MeTHF) = 6.04 g

Amount of the final product: 0.26 g

Amount of waste: 6.04 g – 0.26 g = 5.78 g

E-factor = amount of waste/amount of product = 5.78/0.26 g = **22.1**

E factor for the whole process

Classical Industrial Synthesis

Total amount of reactants: 1.0 g + 0.86 g + 0.096 g + 17.4 g (toluene) + 8.56 g (Et_2O) + 0.504 g + 4.03 g + 2.10 g + 1.15 g + 4.28 g (Et_2O) + 0.326 g + 1.14 g + 2.14 g (Et_2O) + 2.14 g (Et_2O) = 45.73

Amount of the final product: 0.24 g

Amount of waste: 45.73 g – 0.24 g = 45.49 g

E-factor = amount of waste/amount of product = 45.49/0.242 g = **188.0**

Reaction in CPME

Total amount of reactants: 1.0 g + 0.43 g + 0.096 g + 8.6 g (CPME) + 5.16 g (CPME) + 0.504 g + 4.03 g + 2.10 g + 0.72 g + 0.86 g (CPME) + 2.58 g (CPME) 0.326 g + 1.14 g + 0.28 + 4.30 (CPME) = 32.13 g

Amount of the final product: 0.26 g

Amount of waste: 32.13 g – 0.26 g = 31.87 g

E-factor = amount of waste/amount of product = 31.87/0.262 g = **121.6**

Reaction in 2-MeTHF

Total amount of reactants: 1.0 g + 0.43 g + 0.096 g + 8.6 g (2-MeTHF) + 5.16 g (2-MeTHF) + 0.504 g + 4.03 g + 2.10 g + 0.72 g + 0.86 g (2-MeTHF) + 2.58 g (2-MeTHF) 0.326 g + 1.14 g + 0.28 + 4.30 (2-MeTHF) = 32.13 g

Amount of the final product: 0.25 g

Amount of waste: 32.13 g – 0.252 g = 31.88 g

E-factor = amount of waste/amount of product = 31.88/0.252 g = **126.5**

4. Experimental Procedures. α-Functionalization of indanone.

4.1. Table S1. Treatment of 1-indanone (**7**) with different bases and reaction with electrophiles (Scheme 3,a–c, main text).

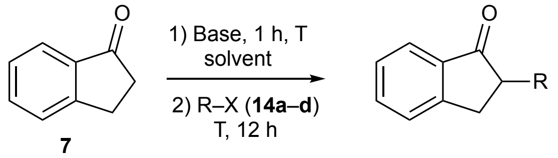

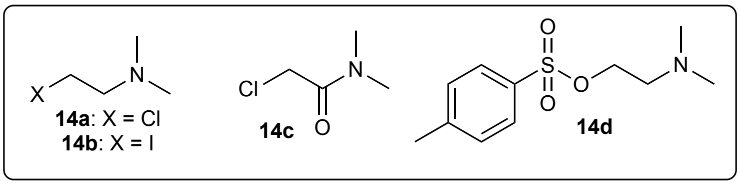

Entry	RX (equiv)	Solvent	Base (equiv)	T (1) °C	T (2) °C	NMR yield%
1	**14a** (1)	THF	LDA (2)	-78	-78	NR[a]
2	**14b** (1)	THF	LDA (2)	-78	-78	NR
3	**14a** (1)	THF	LDA (2)	-78	RT	NR
4	**14a** (1)	THF	KHMDS (2)	-78	RT	NR
5	**14c** (1)	THF	LDA (2)	-78	-78	30[b]
6	**14c** (2)	THF	LDA (2)	-78	0	20
7	**14a** (1.2)	toluene	NaHMDS (2)	-20	0	NR[a]
8	**14c** (2)	CPME	LDA (2)	0	0	20
9	**14c** (1.2)	CPME	LDA (2)	0	0	20
10	**14c** (1)	CPME	LDA (2)	0	0	30
11	**14c** (1)	ChCl/Gly[c]	LDA (2)	RT	RT	NR
12	**14c** (1)	ChCl/urea[c]	LDA (2)	RT	RT	NR
13	**14c** (1)	CPME-ChCl/urea[d]	LDA (2)	0	RT	NR
14	**14c** (1)	CPME	NaH (1)	RT	RT	20[e]
15	**14d** (1.2)	toluene	NaH (1.5)	-20	0	NR
16	**14d** (1.2)	THF	NaH (2)	-78	RT	NR
17	**14d** (1.2)	THF	LDA (2)	0	0	NR
18	**14d** (1.2)	CPME	NaH (1.5)	0	0	NR

Reaction conditions: to a solution of 1-indanone (**7**) (1 mmol) in the proper solvent, the base was added under an Argon atmosphere at T (1). After stirring at T (1) for 1 h, the electrophile was added, and after 12 h stirring at T (2), the reaction was quenched with water. [a] Quenching with MeI: 80% yield. [b] Yield of isolated compound after column chromatography on silica gel. [c] Choline chloride (ChCl)/ glycerol (Gly) (1:2 mol mol^{-1}); ChCl /urea (1:2 mol mol^{-1}). [d] The solution of indanone **7** and LDA in CPME was added to a solution of amide **14c** in DES. [e] The product of bis-addition also formed. RT = room temperature, 25 °C. ^{1}H NMR yield calculated using dibromomethane as an internal standard. NR = no reaction.

4.2. Table S2. Ru-catalyzed hydrogen-borrowing C–C bond formation: reaction between 1-indanone (**7**) and 2-(dimethylamino)ethan-1-ol (**14e**) (Scheme 3,d, main text).

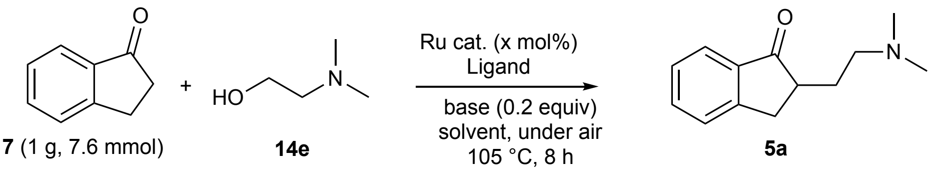

Ru cat.: dichloro(*p*-cymene)ruthenium (II) dimer
Ligand: (oxydi-2,1-phenylene)bis(diphenylphosphine)

Entry	14e (equiv)	Ru cat. mol%	Solvent	Base	Yield % (5a)
1	2	0.5	toluene	NaOH	NR
2	2	0.5	toluene	$CsCO_3$	NR
3	7	0.5	toluene	$CsCO_3$	NR
4	14	0.5	–[a]	$CsCO_3$	NR
5	14	5	–[a]	$CsCO_3$	NR

Reaction conditions: to a solution of **7** (7.6 mmol) and **14e** in toluene (entries 1–3), the catalyst, the ligand and the base were added under air, and the reaction mixture was heated to 105 °C. After 8 h stirring at this temperature, the mixture was cooled to 25 °C. Then, after the addition of diisopropyl ether (5 mL), the mixture was filtered on silica gel. [a] Neat conditions. NR = no reaction.

4.3 Table S3. α-Functionalization of 1-indanone (**7**) via enamine **12** (Scheme 3,e, main text).

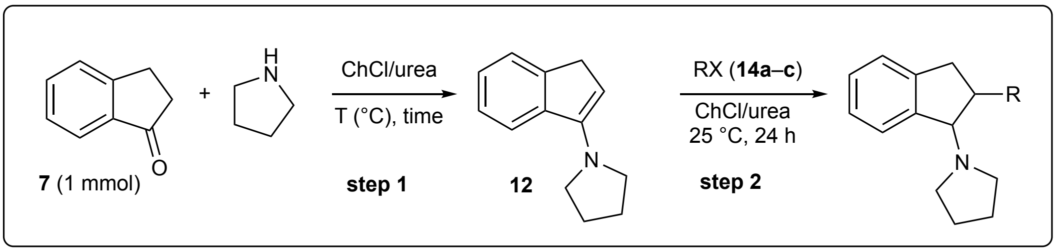

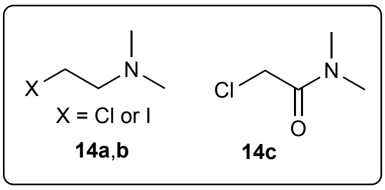

Entry	12 (equiv)	T (°C) (1st step)	Time (h)	NMR yield% (12)
1	2	50	24	NR
2	2	RT	24	80
3	4	RT	2	93

^{1}H NMR yield calculated using dibromomethane as an internal standard. NR = no reaction.

By reacting 1-indanone (**7**) with pyrrolidine (2 equiv), at 50 °C in ChCl/urea for 24 h, no reaction took place (entry 1, Table 4.3). The temperature was lowered to room temperature (RT, 25 °C), and the reaction was monitored via GC for 24 h: adduct **12** was isolated in 80% yield (entry 2, Table 4.3). After adding pyrrolidine (4 equiv) at RT, the desired adduct **12** was isolated in 93% yield after 2 h (entry 3, Table 4.3).

The reaction between 1-indanone (**7**) and pyrrolidine, to give enamine **12**, was run at RT in ChCl/urea, and monitored by GC. After 2 h, amine **14a** or **14b**, or amide **14c** (2 equiv each), was added to the reaction mixture, which was additionally stirred for 24 h: no reaction took place.

4.4. Table S4. Reaction between 2-chloro-*N*,*N*-dimethylethan-1-amine (**14a**) and β-ketoester **13** (Scheme 3,f, main text).

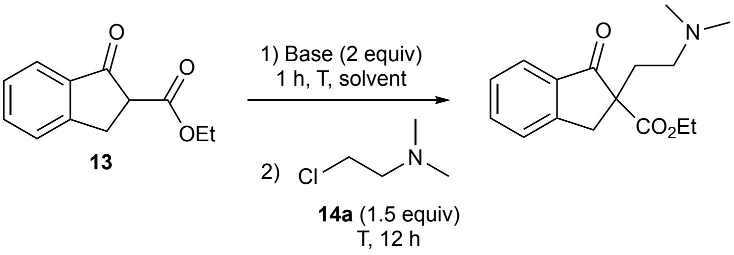

Entry	Solvent	Base	T (1) °C	T (2) °C	Yield %
1	THF	LDA	-78	-78	NR
2	THF	LDA	0	RT	NR
3	THF	KHMDS	-20	RT	NR
4	THF	NaHMDS	-20	RT	NR
5	THF	NaH	-20	RT	NR
6	toluene	NaH	-20	RT	NR
7	CPME	NaH	-20	RT	NR
8	CPME-ChCl/urea[a]	NaH	0	RT	NR
9	CPME-ChCl/urea[a]	LDA	0	RT	NR

Reaction conditions: to a solution of **13** in the proper solvent, the base was added under an Argon atmosphere at T (1). After stirring at T (1) for 1 h, **14a** was added, and the reaction was finally quenched with water after 12 h stirring at T (2). [a] ChCl/urea (1:2 mol mol^{-1}); the solution of ketoester **13** and base in CPME were added to a solution of amine **14a** in DES. NR = no reaction.

5. ^{1}H and ^{13}C NMR spectra

^{1}H NMR, 600 MHz, $CDCl_3$

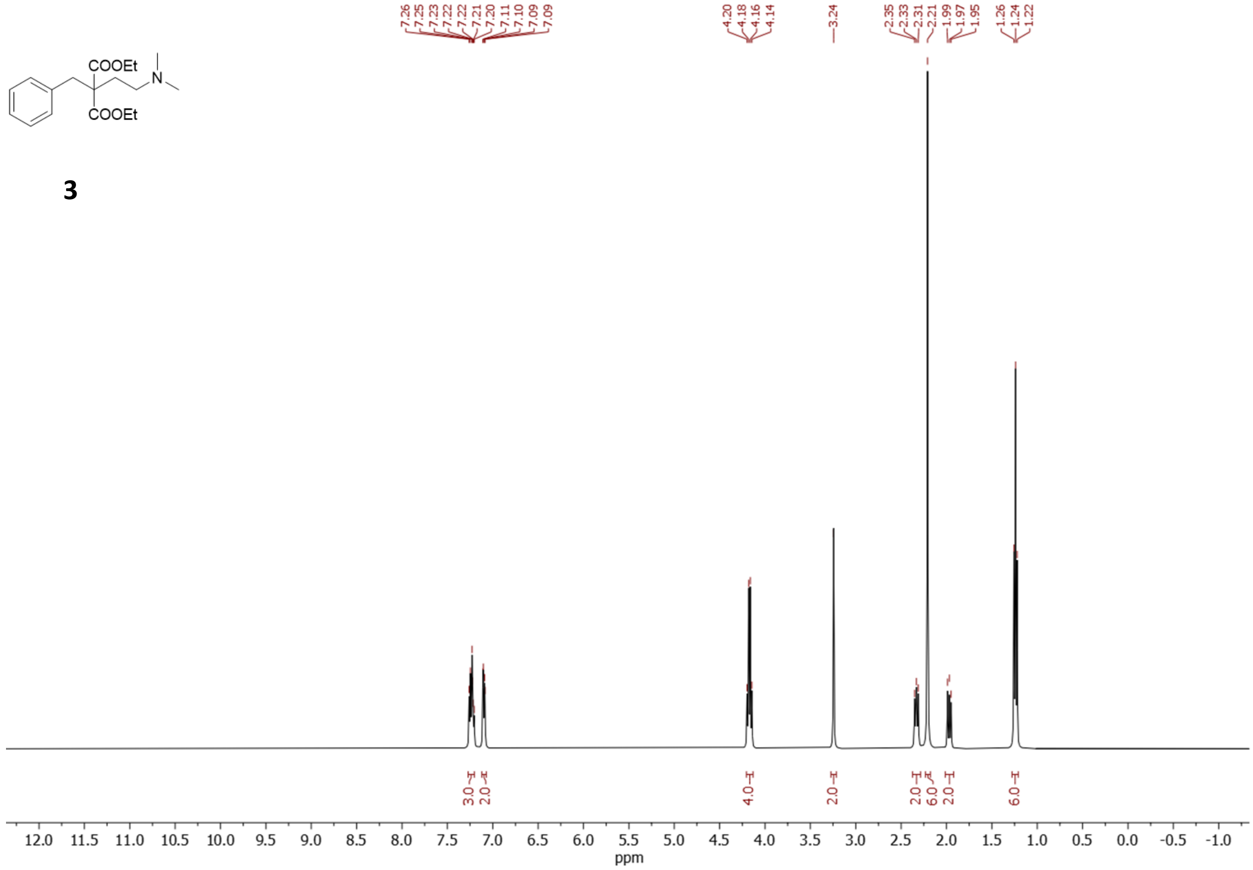

^{13}C NMR, 150 MHz, $CDCl_3$

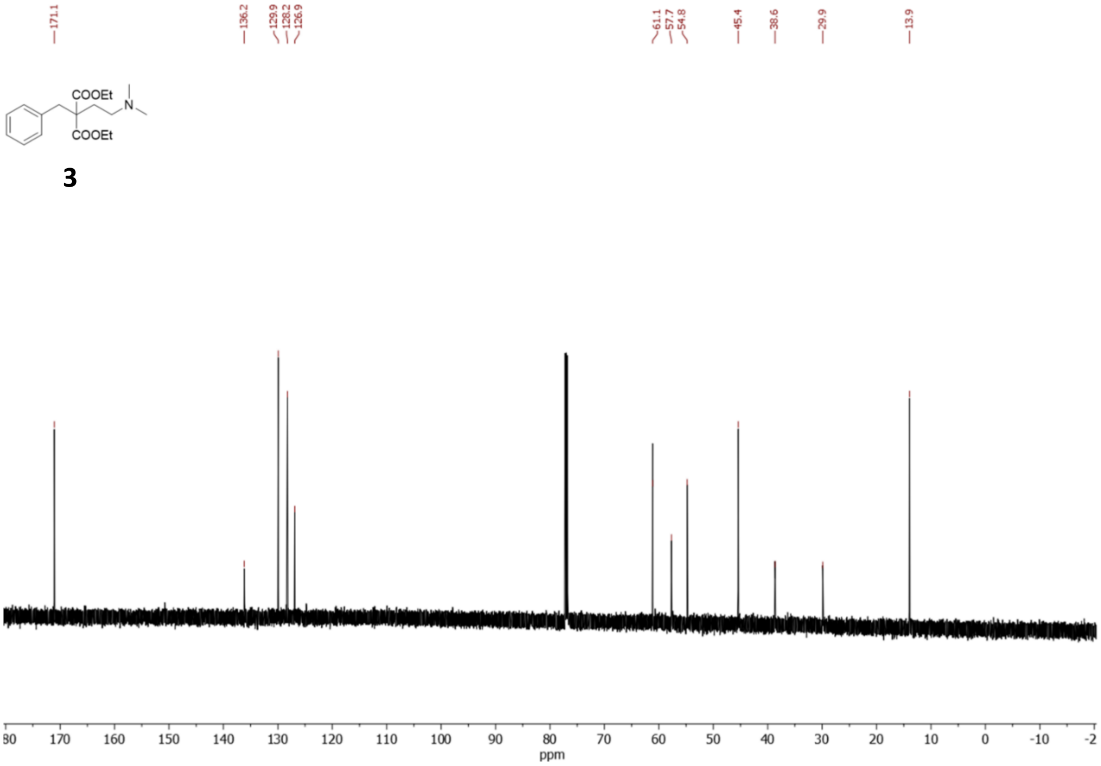

^{1}H NMR, 600 MHz, D_2O

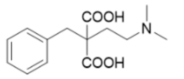

4

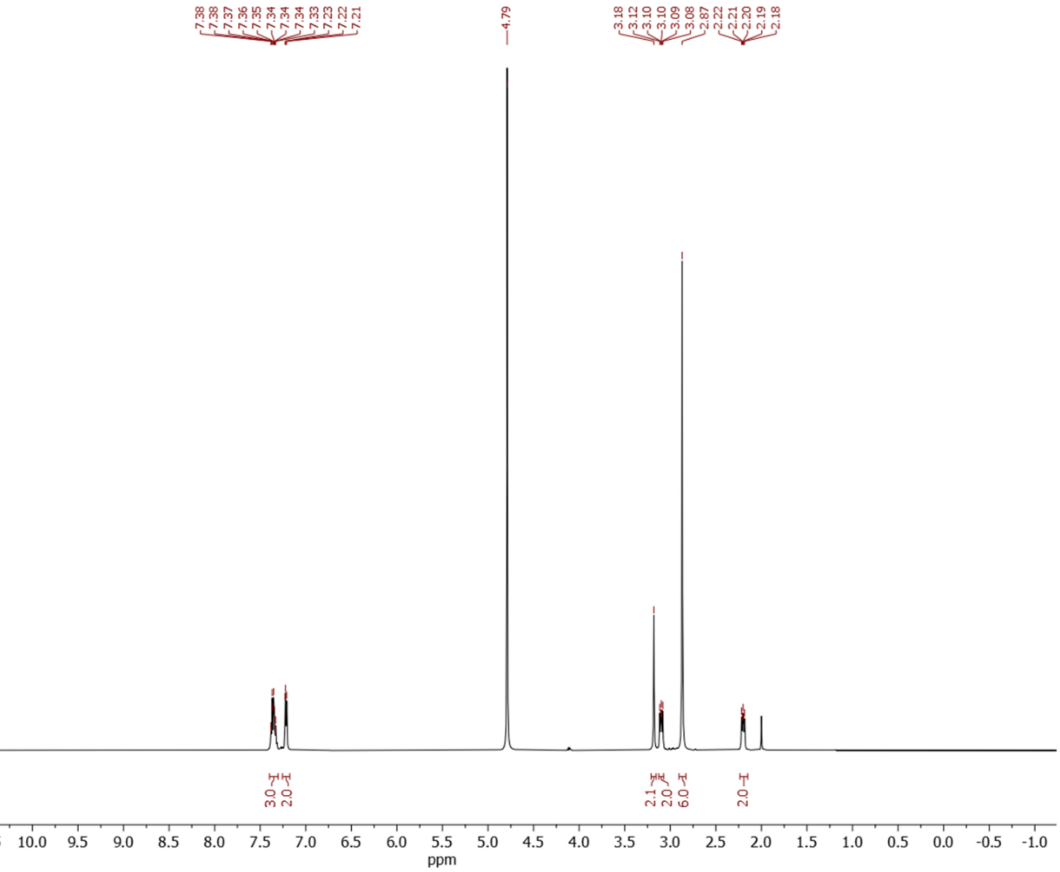

^{13}C NMR, 150 MHz, D_2O

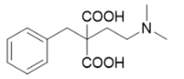

4

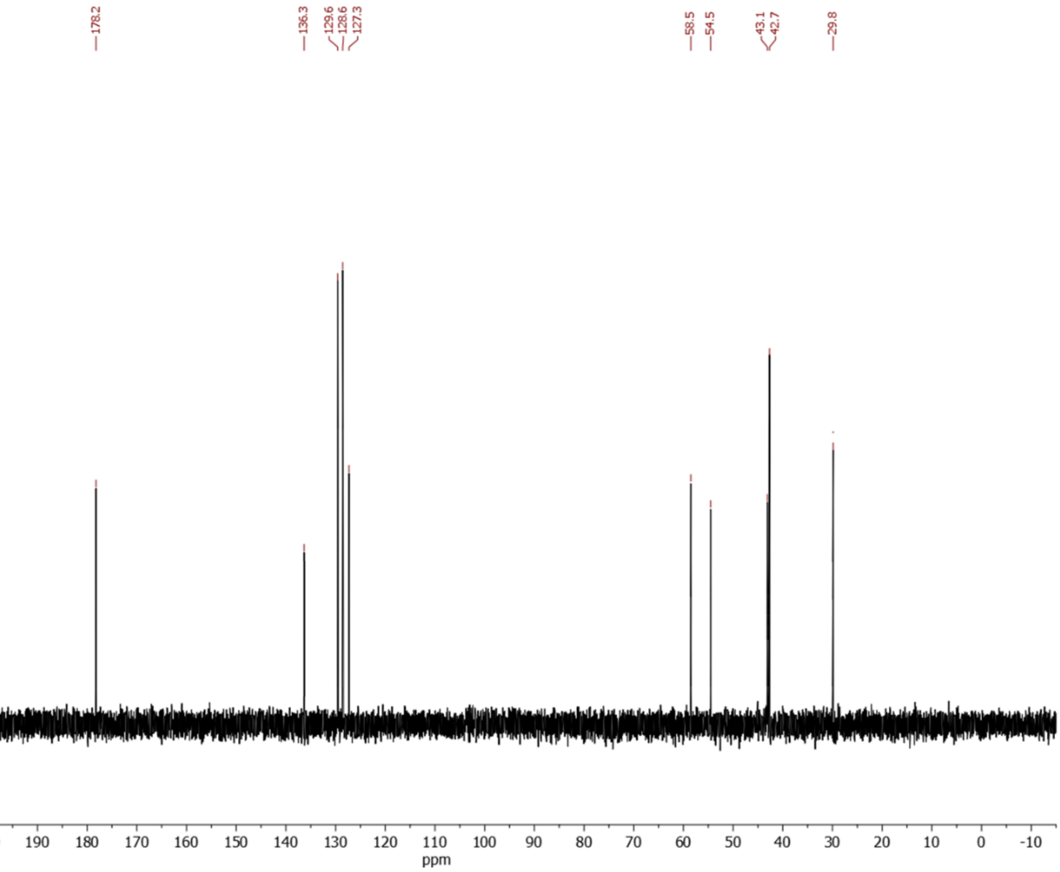

^{1}H NMR, 600 MHz, $CDCl_3$

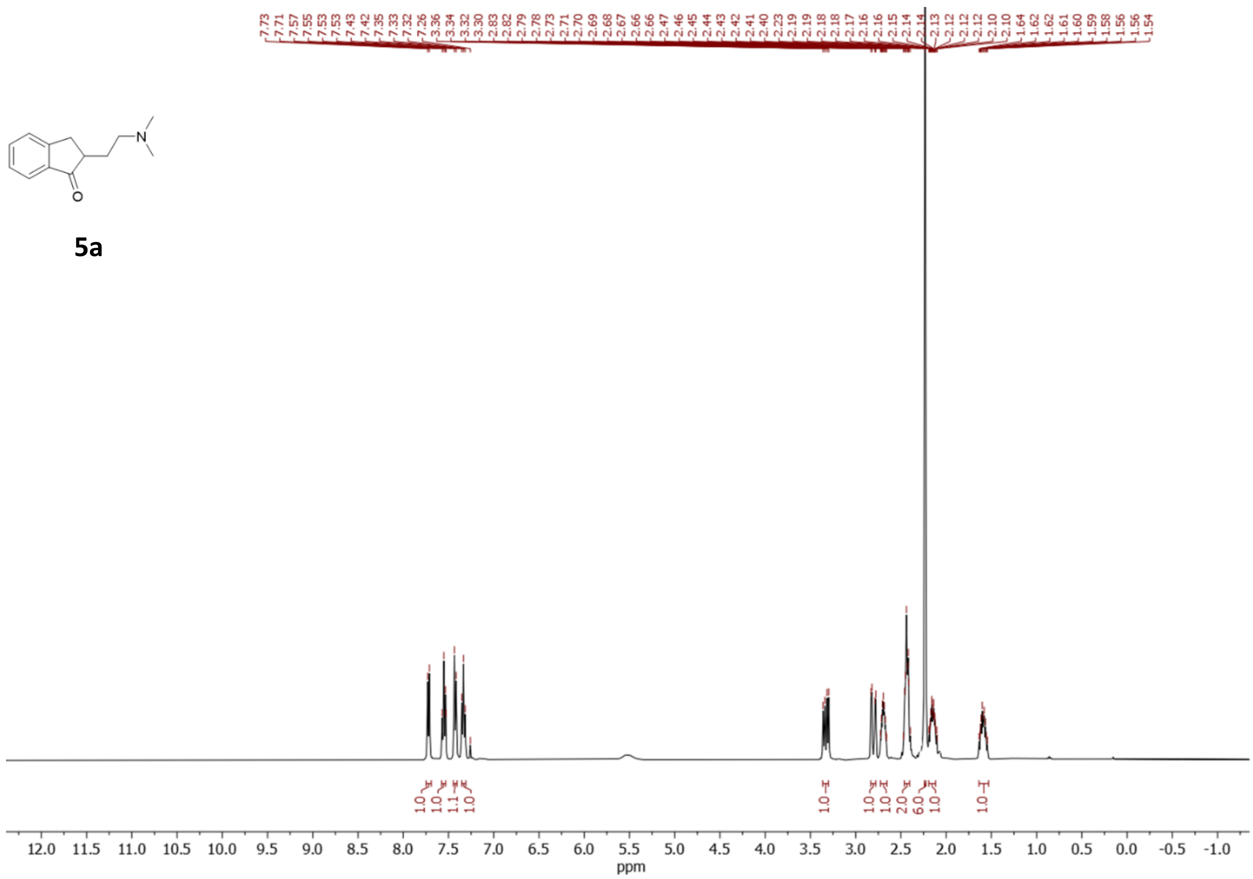

5a

^{13}C NMR, 150 MHz, $CDCl_3$

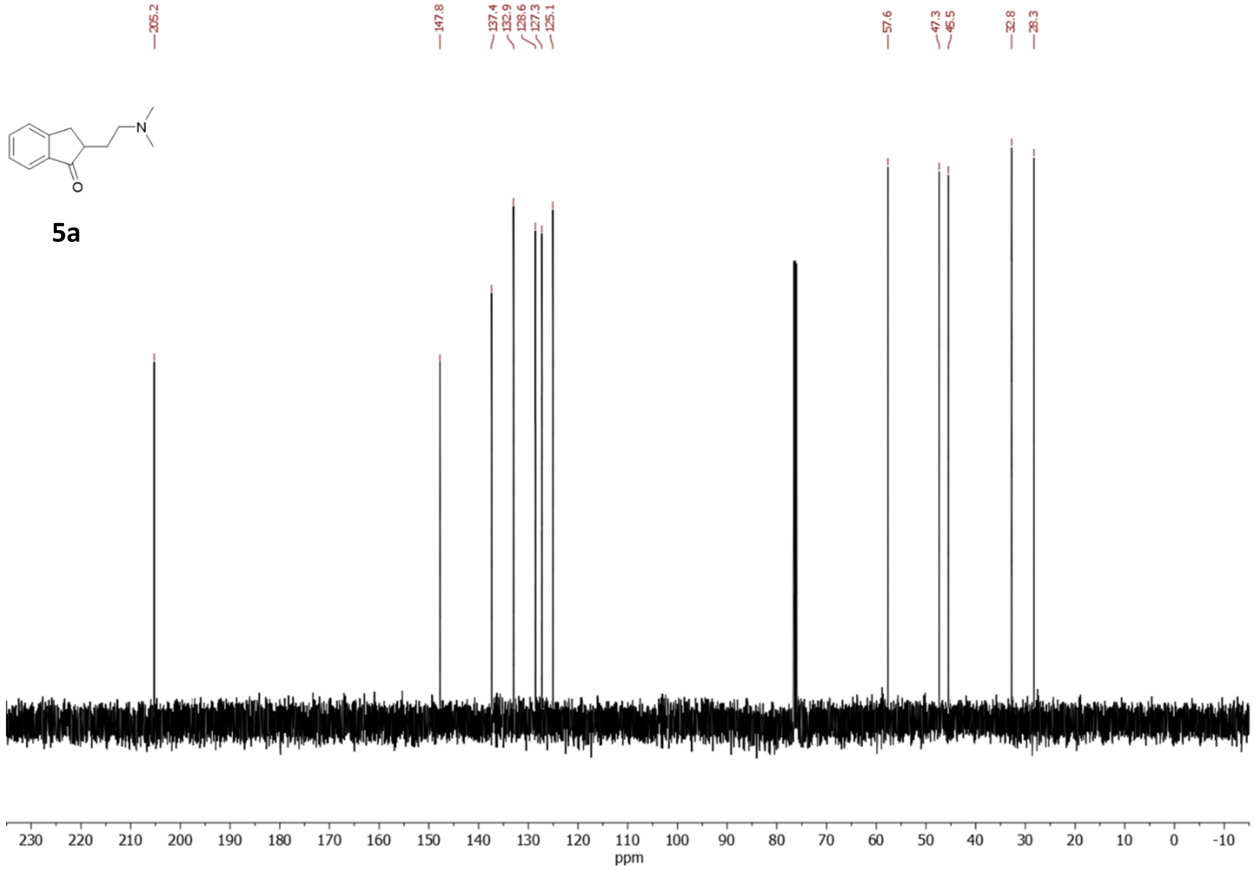

5a

^{1}H NMR, 600 MHz, $CDCl_3$

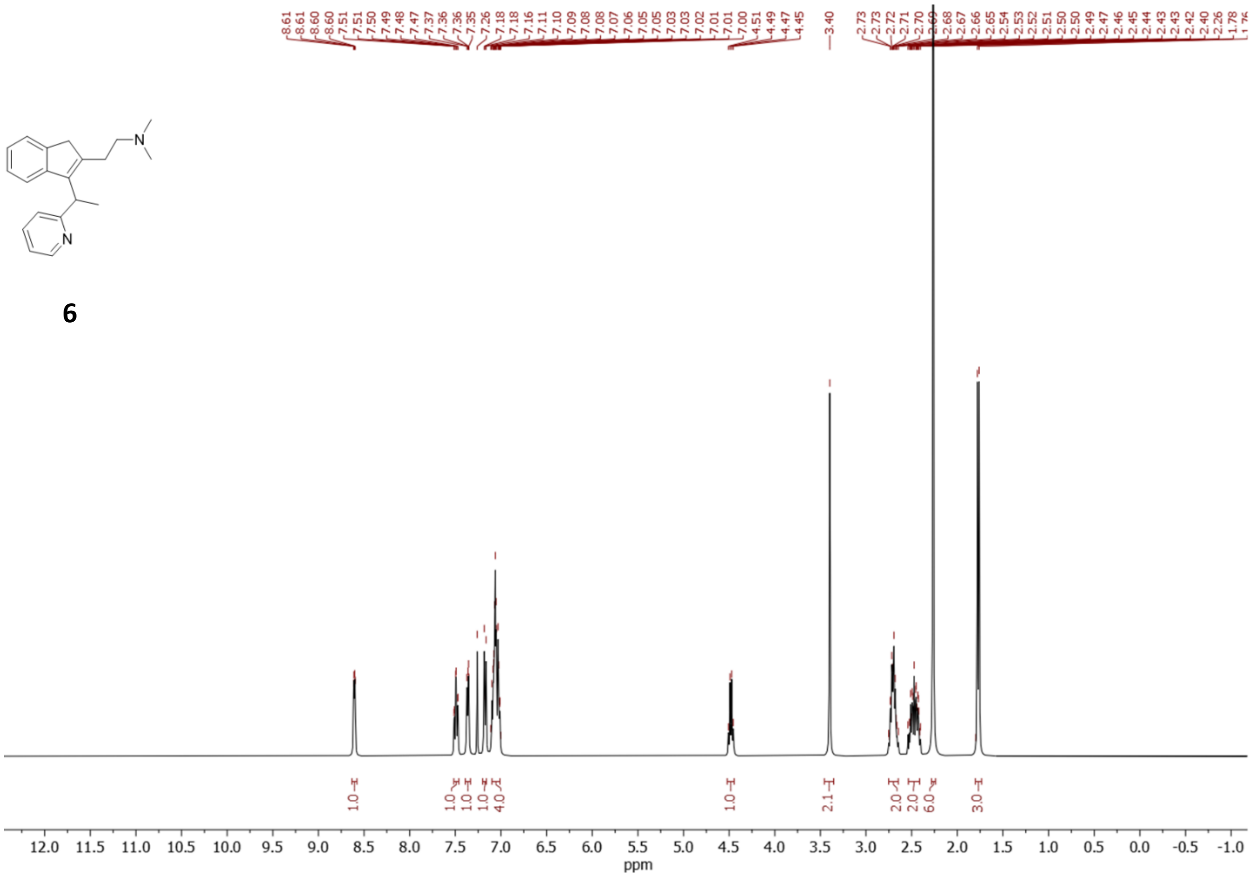

6

^{13}C NMR, 150 MHz, $CDCl_3$

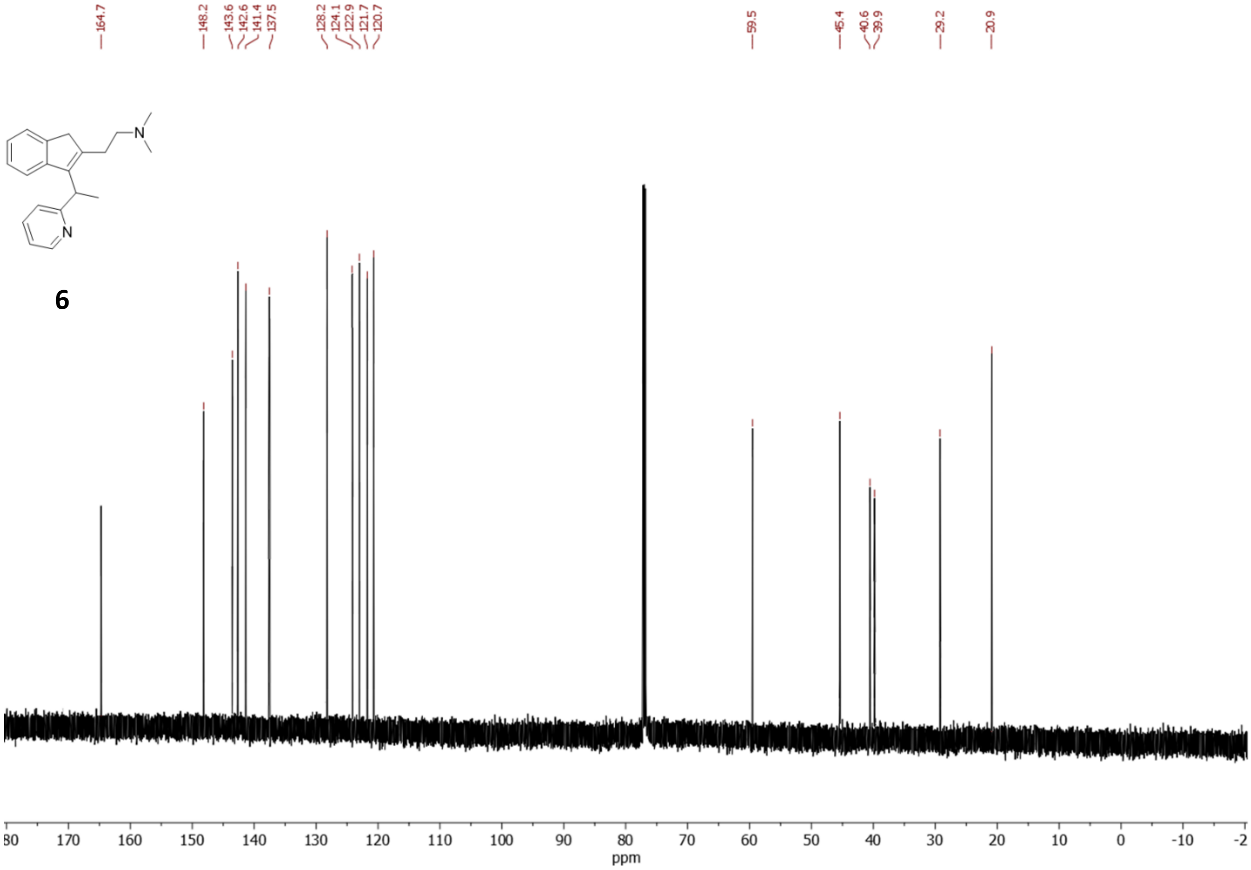

6
